# Forward and Backward Inference in Spatial Cognition

**DOI:** 10.1371/journal.pcbi.1003383

**Published:** 2013-12-12

**Authors:** Will D. Penny, Peter Zeidman, Neil Burgess

**Affiliations:** 1 Wellcome Trust Centre for Neuroimaging, University College, London, London, United Kingdom; 2 Institute for Cognitive Neuroscience, University College, London, London, United Kingdom; École Normale Supérieure, College de France, CNRS, France

## Abstract

This paper shows that the various computations underlying spatial cognition can be implemented using statistical inference in a single probabilistic model. Inference is implemented using a common set of ‘lower-level’ computations involving forward and backward inference over time. For example, to estimate where you are in a known environment, forward inference is used to optimally combine location estimates from path integration with those from sensory input. To decide which way to turn to reach a goal, forward inference is used to compute the likelihood of reaching that goal under each option. To work out which environment you are in, forward inference is used to compute the likelihood of sensory observations under the different hypotheses. For reaching sensory goals that require a chaining together of decisions, forward inference can be used to compute a state trajectory that will lead to that goal, and backward inference to refine the route and estimate control signals that produce the required trajectory. We propose that these computations are reflected in recent findings of pattern replay in the mammalian brain. Specifically, that theta sequences reflect decision making, theta flickering reflects model selection, and remote replay reflects route and motor planning. We also propose a mapping of the above computational processes onto lateral and medial entorhinal cortex and hippocampus.

## Author Summary

The ability of mammals to navigate is well studied, both behaviourally and in terms on the underlying neurophysiology. Navigation is a well studied topic in computational fields such as machine learning and signal processing. However, studies in computational neuroscience, which draw together these findings, have mainly focused on specific navigation tasks such as spatial localisation. In this paper, we propose a single probabilistic model which can support multiple tasks, from working out which environment you are in, to computing a sequence of motor commands that will take you to a sensory goal, such as being warm or viewing a particular object. We describe how these tasks can be implemented using a common set of lower level algorithms that implement ‘forward and backward inference over time’. We relate these algorithms to recent findings in animal electrophysiology, where sequences of hippocampal cell activations are observed before, during or after a navigation task, and these sequences are played either forwards or backwards. Additionally, one function of the hippocampus that is preserved across mammals is that it integrates spatial and non-spatial information, and we propose how the forward and backward inference algorithms naturally map onto this architecture.

## Introduction

This paper describes a dynamic Bayesian model of spatial cognition. Here we define spatial cognition as including the tasks of localisation (estimating where you are in a known environment), sensory imagery (constructing a virtual scene), decision making (deciding which way to turn to reach a goal), model selection (working out which environment you are in) and motor planning (computing a sequence of motor commands that will lead to a sensory goal). We show that all of these tasks can be implemented using statistical inference in a single probabilistic model. We note that the above formulation is slightly different to previous definitions by OKeefe and Nadel [1], Gallistel [2], and Redish [3] which stress the capacity of determining and performing a path from a current position towards a desired location.

The model has hidden states comprising speed, direction and allocentric location, control variables comprising change in direction and speed, and sensory states representing olfactory, somatosensory and visual information. The model describes the dynamical evolution of hidden states, and provides a mapping from hidden to sensory states. Inference in the model is then implemented using a common set of ‘lower-level’ computations involving forward and backward inference over time. We propose that these computations are reflected in recent empirical findings of pattern replay in the mammalian brain [4], [5]. Specifically, we propose that theta sequences reflect decision making, theta flickering reflects model selection, and remote replay reflects route and motor planning. Our use of the terms ‘forward’ and ‘backward’ here relate to time and should not be confused with the direction of message passing in a cortical hierarchy [6].

Our approach falls into the general category of ‘map-based’ or ‘model-based’ planning [1], [7]–[10], or ‘model-based decision making’ [11]. The term ‘model-based’ refers to making and updating a representation of the world (such as a cognitive map). This is to be contrasted, for example, with ‘model-free’ approaches in which agents merely react to stimuli, after having previously learnt stimulus-response mappings through extensive exposure to an environment [12].

More generally, agents will use a variety of navigation strategies depending on their cognitive capabilities and familiarity with an environment. Spatial decisions can, for example, be classified [13] as being cue-guided (eg. move towards the red house), stimulus triggered (eg. turn left at the red house), route based (turn left at the red house then right at the blue house). There is a good deal of evidence showing that the brain has multiple decision making or control systems, each with its own strengths and weaknesses [14]–[16].

The usefulness of model-based planning is most apparent after an agent has sufficient experience to learn a model of an environment and when, subsequently, local changes to that environment are made which affect the optimal route to a goal [15]. In statistical terms, these would be referred to as nonstationarities. For spatial models this could be, for example, a hole appearing in a wall enabling an agent to take a shortcut, or a new object appearing preventing an agent taking a habitual route. Another strength of model-based control is that it can reduce learning time. Tse et al. [17], for example, studied decision making in rats and found that learning required fewer trials when it occurred against a background of prior knowledge. This allows new information to be assimilated into an existing schema or model.

The model-based versus model-free distinction has become important for the study of decision making in general as the underlying neuroanatomical differences are being delineated [11], [15]. Khamassi and Humphries [18] argue that, due to the shared underlying neuroanatomy, spatial navigation strategies that were previously described as being either place-driven or cue-driven are better thought of as being model-based versus model-free. Daw et al. [15] propose that arbitration between model-based and model-free controllers is based on the relative uncertainty of the decisions and more recently, Pezzulo et al. [19] have embedded both types of decision making systems into a single ‘mixed instrumental controller’.

This paper describes the computations underlying spatial cognition, initially, at a rather abstract level of manipulations of probability densities and then employs vector and matrix representations of variables and connectivities. Although we later on go on to describe how our model relates to underlying neuronal implementations, the model itself is not specified at a neuronal level. This style of modelling has many precedents in the literature. For example, Bousquet et al. [20] have conceived of the hippocampus as a Kalman filter. This requires that the hippocampus has an ‘observation model’ relating hidden states (places specified in allocentric coordinates) to sensory cues, and a dynamic model relating previous to current state via path integration. Kalman filtering then refers to the forward inference algorithm that combines path integral estimates of state with current sensory cues to provide optimal updates of the agent's location. The main function of Kalman filtering in this context is therefore one of localisation. One of the key points of this paper is that if an agent has taken the trouble to construct a ‘dynamic model’ and an ‘observation model’ then they can be used for more than just localisation; the same models, when combined with additional inference steps, can also be used for model selection, decision making and motor planning and to construct sensory imagery.

Other statistical treatments of hippocampal function address the issue of context learning [21]. Here, a context is defined in statistical terms as a stationary distribution of experiences. The problem of context learning is then reduced to one of clustering together an agent's experiences into a finite number of contexts. This is addressed through the use of Hidden Markov Models (HMMs) and it is shown how this perspective explains experimental findings in rat navigation concerning sequence and reversal learning and place-cell remapping. Johnson et al. [22] provide a normative statistical model of exploratory behaviour called Information Foraging (IF). ‘Passive IF’ describes the temporal distribution of an agent's sampling process (eg. spending longer investigating novel versus familiar objects) whereas ‘Directed IF’ describes its spatial distribution (eg. where it should move to next). Additionally, IF is conceived to apply both to the environment and the agent's memory of the environment. Directed IF proposes a common hippocampal substrate for constructive memory (eg. scene construction), vicarious trial and error behaviour, model-based facilitation of memory performance, and memory consolidation. The IF framework samples spatial locations, or episodic memories using an information theoretic criterion. To compute this criterion it is necessary for the agent to possess an observation model of the sort described in our article below. A further statistical treatment of hippocampal function comprises a two-stage processing model of memory formation in the entorhinal-hippocampal loop [23]. The first stage, which is proposed to take place during theta activity, allows hippocampus to temporally decorrelate and sparsify its input, and develop representations based on an Independent Component Analysis. The second stage, which is proposed to take place during Sharp Wave Ripples [24], allows hippocampus to replay these new representations to neocortex where long term memories are held to be instantiated.

This paper is concerned with computational processes underlying spatial cognition and we describe how the underlying computations may be instantiated in hippocampus and associated brain regions. The hippocampal formation is, however, implicated in a much broader array of functions [25], such as episodic memory, that our model does not address. Indeed one of the key differences between our approach and some other models of spatial cognition [10], [16] is that the approach we describe has no episodic component. Specifically, the sequences that are generated in our model are the result of online computation rather than memory recall. However, as we highlight in the discussion, the interactions between episodic memory and the computations we describe would be especially interesting to examine in future work.

The paper is structured as follows. The computer simulations in this paper describe an agent acting in a simple two-dimensional environment. This environment produces visual, somatosensory and olfactory cues as described in the methods section on the ‘Environmental Model’. The agent then develops its own model of the environment as described in the ‘Probabilistic Model’ section. This describes the two elements of the model (i) a dynamical model describing the evolution of hidden states and (ii) a mapping from hidden states to sensory states. The section on ‘Spatial Cognition as Statistical Inference’ then describes how the various tasks of localisation, decision making (and sensory imagery), model selection and motor planning can be described in probabilistic terms. The section on ‘Forward and Backward Inference’ describes the common set of forward and backward recursions for estimating the required probability densities. The section on ‘Results’ describes an implementation of the above algorithms and provides some numerical results. The discussion section on ‘Neuronal Implementation’ then describes our proposal for how these algorithms are implemented in the brain and how functional connectivity among a candidate set of brain regions changes as a function of task. We conclude with a discussion of how the above computations might relate to pattern replay and what are the specific predictions of our model.

## Methods

In what follows matrices are written in upper case bold type and vectors in lower case bold. Scalars are written in upper or lower case plain type. We use 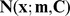 to denote a multivariate Gaussian density over the random variable 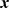 having mean 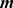 and covariance 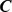. Table 1 provides a list of all the symbols used in the main text.

**Table 1:** Description of mathematical symbols used in the main text.

**Environmental Model**	
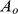	Scaling of olfactory source
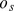	Allocentric location of olfactory source
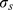	Spatial diffusion of olfactory source
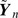	Sequence of sensory states from environmental model
**Sensory State Variables**	
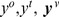	Olfactory, somatosensory and visual states
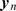	Sensory state (comprising 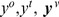 )
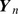	Sequence of sensory states up to time 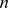 (observations or goals)
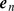	Sensory noise
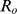	Variance of olfactory noise
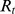	Variance of somatosensory noise
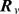	Covariance of visual noise
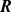	Sensory noise covariance (blkdiag( 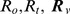 ))
**Control Variables**	
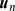	Control signal (virtual input or motor efference copy)
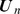	Sequence of control signals up to time index 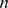
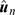	Estimate of control signal from backward inference
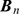	Uncertainty in est. of control signal from backward inference
**Hidden State Variables**	
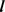	Allocentric location comprising 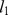 and 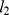
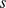	Speed
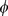	Direction of heading
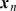	Hidden state (comprising 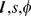 ) at time step 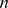
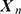	Hidden state sequence up to time index 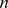
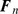	Flow term describing change of state wrt. previous state
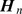	Flow term describing change of state wrt. input
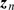	Hidden state noise
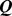	Hidden state noise covariance
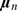	State estimate from path integration (forward inference)
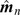	State estimate based on Bayes rule (forward inference)
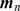	State estimate from backward inference
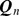	Covariance of state estimate from path integration
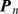	Covariance of state estimate from Bayes rule (forward inference)
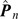	Covariance of state estimate from backward inference
**Agent's Observation Model**	
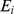	Model of environment i
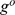 , 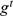 , 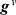	Agent's predictions of olfactory, somatosensory and visual state
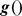	Agent's predictions of sensory state
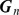	Local linearisation of observation model
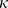	Precision of head direction cells
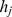	Output of 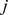 th head direction cell
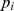	Output of 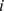 th spatial basis function
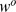 , 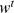 , 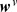	Weights in agent's olfactory, somatosensory and visual models

### Environmental Model

**Figure 1:**
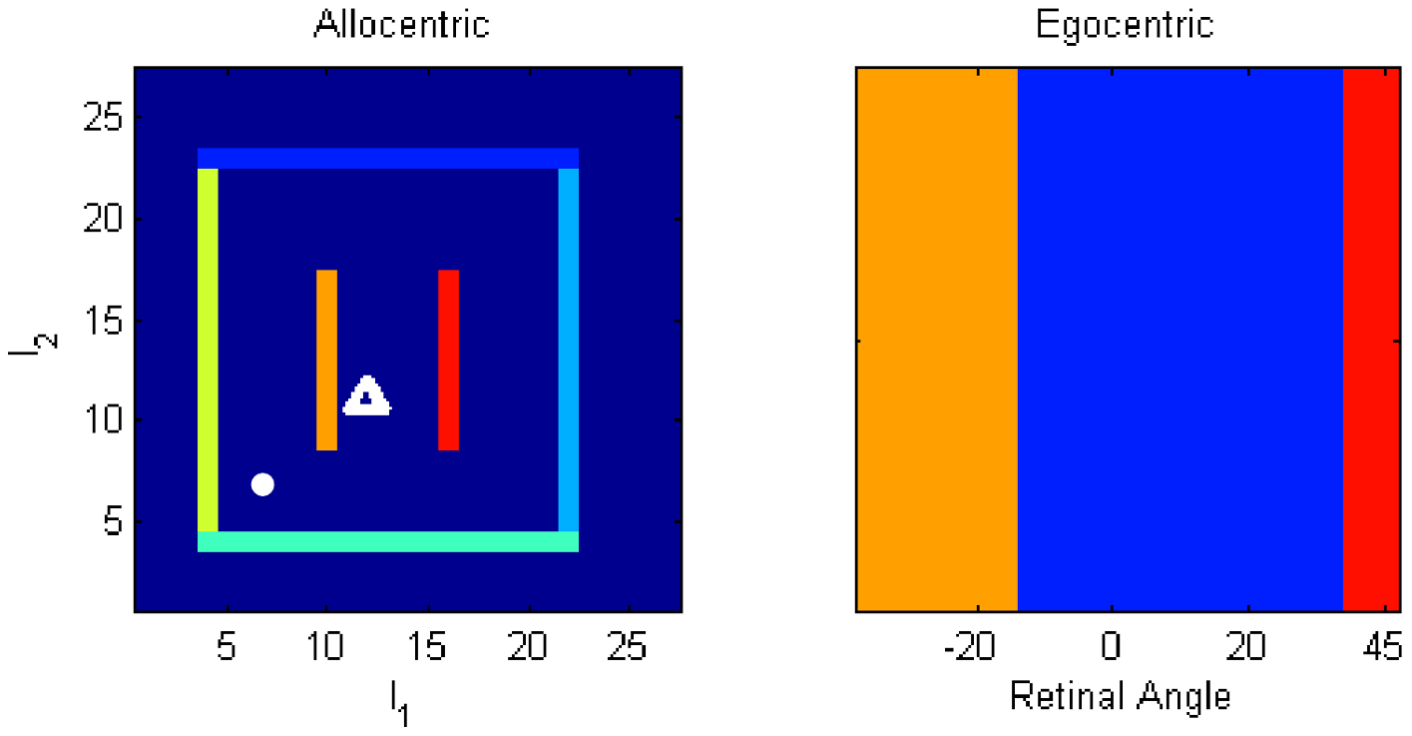
Model of environment. Allocentric representation (left panel) and egocentric view (right panel). The agent (white triangle) is at allocentric location 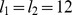 and oriented at 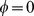 degrees (clockwise relative to the positive 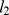 axis). The environment contains two inner walls and four boundary walls. The agent is equipped with whiskers that detect the minimum Euclidian distance to a wall, 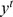. It is also equipped with a nose that detects the signal from an olfactory source placed at 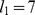, 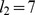 in the south-west corner of the maze (white circle). The agent also has a retina that is fixed in orientation and always aligned with the direction of heading, 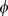. The retina provides one-dimensional visual input, 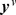 (displayed as a one-dimensional image in the right panel), from −45 to +45 degrees of visual angle around 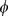 and comprising 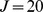 pixels.

Computer simulations are implemented in Matlab (R2012a, The MathWorks, Inc.) and are based on an agent navigating in a simple 2D environment depicted in Figure 1. The location of the agent is specified using orthogonal allocentric coordinates 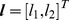 and its direction of heading (clockwise from positive 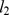) is 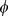. The environment contains two inner walls and four boundary walls. The agent is equipped with a touch sensor that detects the minimum Euclidian distance to a wall, 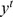. It is also equipped with a nose that detects olfactory input, 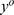. In this paper we consider a single olfactory source located at allocentric coordinates 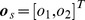. We assume this source diffuses isotropically with scale parameter 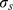 so that olfactory input at location 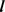 is given by an exponential function
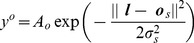
(1)All of the simulations use a single olfactory source with 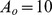, 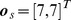 and 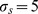. More realistic environments with multiple olfactory sources and turbulence [26] are beyond the scope of this paper.

The agent is also equipped with a retina that is aligned with the direction of heading. The retina provides one-dimensional visual input, 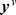, from −45 to +45 degrees of visual angle around 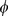 and comprises 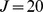 pixels. The retina provides information about the ‘colour’ of the walls within its field of view. In our simulations ‘colour’ is a scalar variable which we have displayed using colormaps for ease of visualisation. The scalar values corresponding to the various walls are 0.14 (north border), 0.29 (east border), 0.43 (south border), 0.57 (west border), 0.71 (west wall), 0.86 (east wall). These map onto the colours shown in Figure 1 using Matlab's default colour map. Although classical laboratory navigation tasks do not involve walls with different colours, they employ extra-maze cues which enable experimental subjects to localize themselves. For the sake of simplicity, here we provide such visual information to the simulated agent by variation of wall colour.

The environmental model of retinal input takes the values of 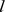 and 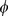 and produces 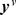 using calculations based on the two-dimensional geometrical relation of the agent with the environment. This uses a simple ray-tracing algorithm. The agent then has its own predictive model of retinal input, described in the ‘vision’ section below, which predicts 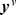 from 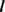 and 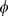 using a basis set expansion. The agent has similar models of olfactory and somatosensory input (see ‘Olfaction’ and ‘Touch’ below). Overall, the environmental model produces the signals 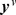, 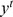 and 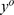 which form the sensory inputs to the agent's spatial cognition model (see next section). We write this as 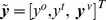 to denote sensory signals from the environment. For a sequence of signals we write 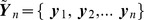. These sensory inputs are surrogates for the compact codes produced by predictive coding in sensory cortices [27]. We emphasise that the agent has its own model of sensory input (an ‘observation model’) which is distinct from the environmental input itself. The agent's observation model is learnt from exposure to the environment.

### Probabilistic Model

**Figure 2:**
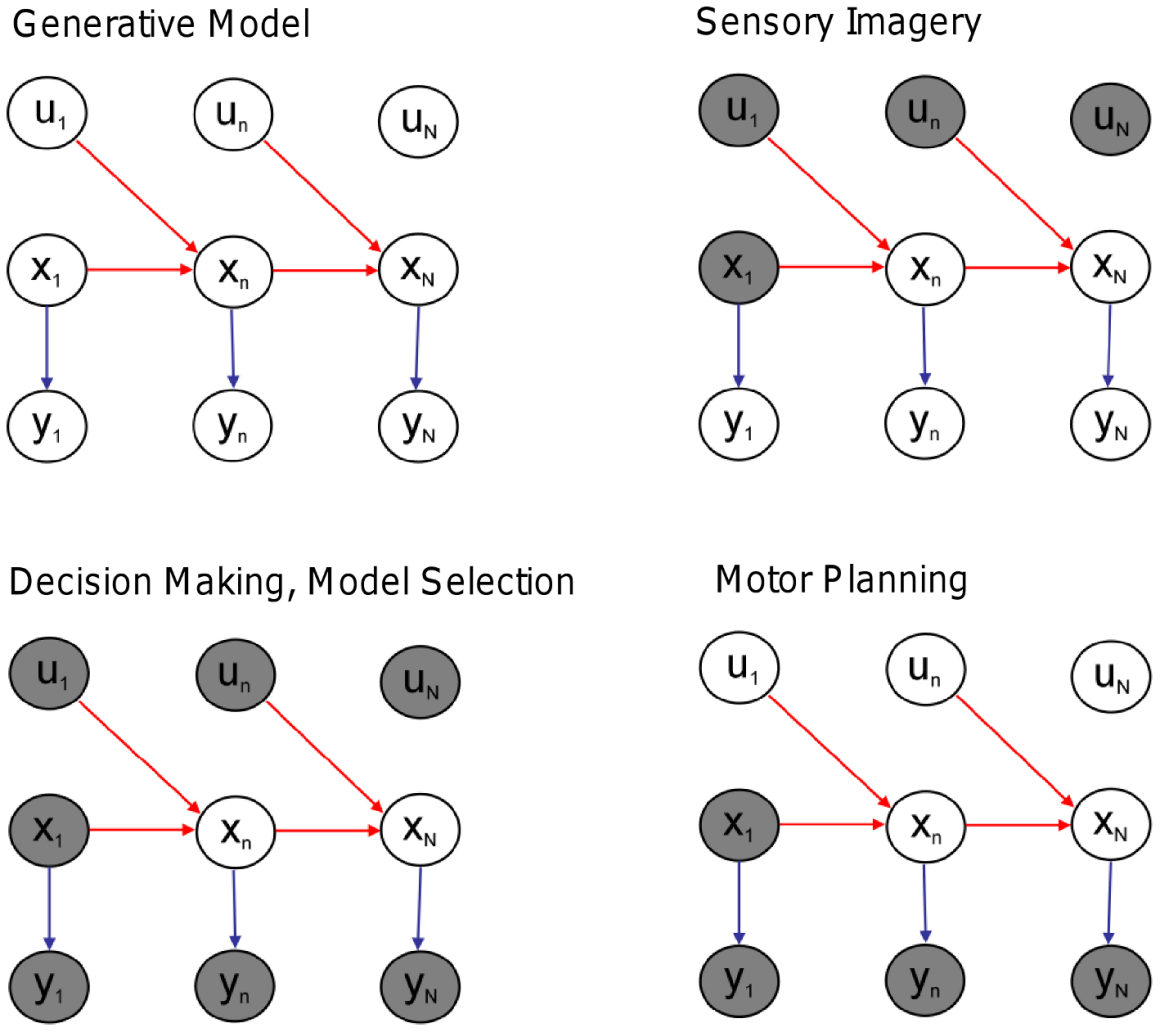
Generative model for spatial cognition. The agent's dynamical model is embodied in the red arrows, 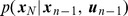, and its observation model in the blue arrows, 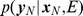. All of the agent's spatial computations are based on statistical inference in this same probabilistic generative model. The computations are defined by what variables are known (gray shading) and what the agent wishes to estimate. **Sensory Imagery** Given a known initial state, 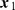, and virtual motor commands 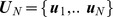, the agent can generate sensory imagery 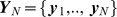. **Decision Making** Given initial state 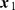, a sequence of putative motor commands 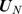 (eg. left turn), and sensory goals 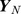, an agent can compute the likelihood of attaining those goals given 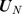 and 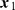, 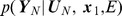. This computation requires a single sweep of forward inference. The agent can then repeat this for a second putative motor sequence (eg. right turn), and decide which turn to take based on the likelihood ratio. **Model Selection** Here, the agent has made observations 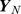 and computes the likelihood ratio under two different models of the environment. **Planning** can be formulated as estimation of a density over actions 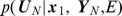 given current state 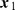 and desired sensory states, 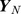. This requires a forward sweep to compute the hidden states that are commensurate with the goals, and a backward sweep to compute the motor commands that will produce the required hidden state trajectory.

We investigate agents having a model comprising two parts (i) a dynamical model and (ii) an observation model. The dynamical model describes how the agent's internal state, 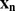 is updated from the previous time step 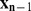 and motor efference copy 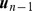. The observation model is a mapping from hidden states 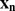 to sensory states 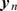. Our probabilistic model falls into the general class of discrete-time nonlinear state-space models
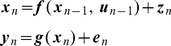
(2)where 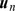 is a control input, 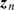 is state noise and 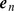 is sensory noise. The noise components are Gaussian distributed with 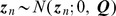 and 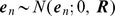. This is a Nonlinear Dynamical System (NDS) with inputs and hidden variables. We consider a series of time points 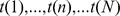 and denote sequences of sensory states, hidden states, and controls using 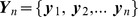, 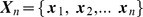, and 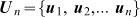. These are also referred to as trajectories. The above equations implicitly specify the state transition probability density 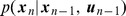 and the observation probability density 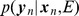. This latter probability depends on the agent's model of its environment, 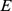. Together these densities comprise the agent's generative model, as depicted in Figure 2 (top left).

#### Path integration

During spatial localisation, an agent's current location can be computed using path integration. This takes the previous location, direction of heading, velocity and elapsed time and uses them to compute current position, by integrating the associated differential equation. We assume that the agent is in receipt of a control signal 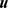 which delivers instructions to change direction, 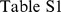, and speed, 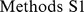. During navigation, for example, these signals will correspond to motor efference copy. Later we will show how these control signals can be inferred by conditioning on desirable future events (i.e. how the agent performs planning). For the moment we assume the controls are known. The dynamical model is
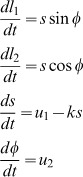
(3)Here the state variables are two orthogonal axes of allocentric location, 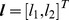, speed 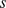 and direction 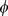 (clockwise angle relative to the positive 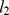 axis). Motion is also subject to frictional forces as defined by the constant 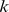. We set 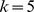. We can write a state vector 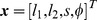. The control signals 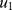 and 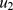 change the agent's speed and direction. We can write
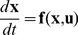
(4)which can be integrated to form a discrete-time representation
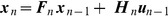
(5)using local linearisation as described in Text S1. If the deterministic component of the dynamics is originally described using differential equations, the flow terms 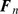 and 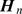 can be computed as shown in Text S1. Here 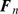 describes how the current hidden state depends on the previous hidden state, and 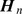 how it depends on the previous input. An example of using the above equations for implementing path integration is described in the ‘Sensory Imagery’ simulation section below. Errors in path integration, perhaps due to inaccuracies in the representation of time or in local linearisation, can also be included, i.e.
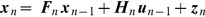
(6)where 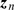 is a random variable. This corresponds to a locally linearised version of equation 2. For the results in this paper we used a local regression method, due to Schaal et al. [28], to compute 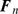 and 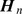 as this resulted in more robust estimates. This is described in Text S1.

#### Multisensory input

We consider agents with sensory states, 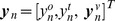 having olfactory, somatosensory and visual components. Sensory states will typically be low-dimensional codes that index richer multimodal representations in sensory cortices. During navigation and model selection these will correspond to inputs from the environmental model, 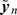. During decision making and motor planning these will correspond to internally generated sensory goals. The agent associates hidden states with sensory states using the mapping 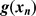, a nonlinear function of the state variables. We have
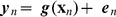
(7)where 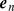 is zero-mean Gaussian noise with covariance 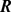. During localisation and model selection 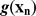 corresponds to the agent's prediction of its sensory input, and 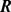 specifies the covariance of the prediction errors. These predictions can be split into modality-specific components 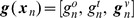 with associated prediction errors having (co-)variances 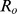, 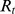 and 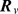. Equation 7 defines the likelihood
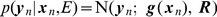
(8)We assume the different modalities are independent given the state so that
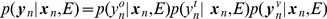
(9)where
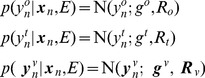
(10)so that 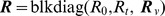. We now describe the agent's model for generating the predictions 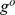, 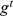 and 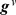. Olfactory input is predicted using a basis set
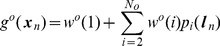
(11)where 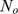 is the number of basis functions, 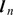 is the location, and 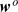 are parameters of the olfactory model. Here we use a local basis function representation where
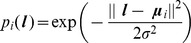
is the response of the 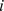
th basis cell. Following Foster et al. [29] 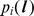 may be viewed as an idealised place cell output, where 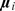 is the spatial location of the centre of cell i's place field, and 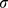 its breadth. We assume that the parameters governing the location and width of these cells have been set in a previous learning phase. In this paper we used 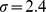 and the centres of the place fields 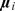 were arranged to form a 10-by-10 grid in allocentric space. The same set of cells were used as a basis for predicting olfactory, somatosensory and visual input.

The parameters 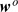 will have to be learnt for each new environment. For the results in this paper they are learnt using a regression approach, which assumes knowledge of the agent's location. More generally, they will have to be learnt without such knowledge and on a slower time scale than (or after learning of) the place cell centres and widths. This is perfectly feasible but beyond the scope of the current paper. We return to this issue in the discussion.

In the agent's model, somatosensory input is predicted using a basis set
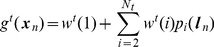
(12)where 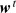 are the parameters of the somatosensory model. Here we envisage that processing in somatosensory cortex is sufficiently sophisticated to deliver a signal 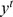 that is the minimum distance to a physical boundary. If the agent had whiskers, a simple function of 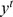 would correspond to the amount of whisker-related neural activity. More sophisticated generative models of somatosensory input would have a directional, and perhaps a dynamic component. But this is beyond the scope of the current paper.

The agent's retina is aligned with the direction of heading, 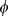. The retina provides one-dimensional visual input, 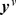, from −45 to +45 degrees of visual angle around 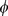 and comprising 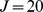 pixels. An example of retinal input is shown in the right panel of Figure 1. The agent's prediction of this visual input is provided by a weighted conjunction of inputs from populations of place/grid and head direction cells. The head direction cells are defined as
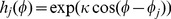
(13)where 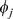 is the preferred angle of the 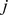
th basis function and 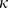 defines the range of angles to which it is sensitive. The output for retinal angle 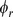 is given simply by 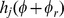. Visual input at retinal angle 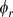 is then predicted to be
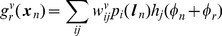
(14)This sort of conjunctive representation is widely used to provide transformations among coordinate systems and, for sensorimotor transforms, is thought to be supported by parietal cortex [30]. The above mapping is adaptable and can be optimised by choosing appropriate weights 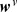 and these will have to be learnt for each new environment.

It is a gross simplification to predict retinal input, or egocentric views, with a single stage of computation as in the above equation. More realistic models of this process [31], [32] propose separate representations of the spatial and textural components of landmarks, with bilateral connectivity to cells in a parietal network which effect a transform between allocentric and egocentric coordinates. Egocentric view cells are then also connected to this parietal network. This level of detail is omitted from our current model, as our aim is to focus on temporal dynamics.

Overall, the agent's model of multisensory input has parameters 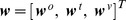. For each new environment, 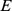, the agent has a separate set of parameters. Experiments on rats have found that changes to the environment cause changes in the pattern of firing of place cells [33], [34]. This could happen in our model if the cells fire at rates 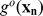, 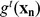 and 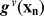 and the parameters 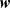 are updated to reflect changes in sensory features. In the simulations that follow the 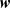 parameters are set using a separate learning phase prior to spatial cognition. More detailed models of this learning process propose that cells in the dentate gyrus select which CA3 cells will be engaged for encoding a new environment [35]. Connections from EC to selected CA3 cells are then updated to learn the relevant place-landmark associations.

### Spatial Cognition as Statistical Inference

This section describes, initially at the level of manipulations of probability densities, how the various computations underlying spatial cognition can be implemented. It then describes a practical algorithm based on local linearisation. If an agent has a probabilistic model of its environment, 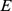, then the various tasks that together comprise spatial cognition are optimally implemented using statistical inference in that model. These inferences will be optimal in the sense of maximising likelihood. The various tasks - localisation, imagery, decision making, model selection and planning - all rely on the same statistical model. They are differentiated by what variables are known and what the agent wishes to compute. This is depicted in the panels in Figure 2 where shaded circles denote known quantities. Additionally, for each task, the information entering the system may be of a different nature. For example, for imagery, the inputs, 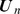, are virtual motor commands and for localisation they are motor efference copies. Similarly, during localisation and model selection the agent receives inputs from sensory cortices. For the simulations in this paper these come from the environmental model, 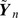. However, during decision making and motor planning these inputs do not derive from the agent's environment but are generated internally and correspond to the agent's goals 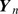.

#### Localisation

The use of dynamic models with hidden states for spatial localisation is well established in the literature [20], [36], [37]. Estimation of spatial location requires motor efference copy 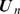, and sensory input 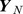. The initial location 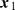 may be known or specified with some degree of uncertainty. Forward inference over states (in time) can then be used to optimally combine probabilistic path integration with sensory input to estimate location. This produces the density 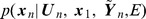. A Gaussian approximation to this density based on a local linearisation is described below in the section on forward inference over states (see equation 24). The agent's best estimate of its location is then given by the maximum likelihood estimate
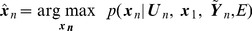
(15)We refer to this as a maximum likelihood estimate because there is no distribution over 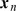 prior to observing the sequence 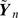. This is commensurate with standard terminology [38]. However, one could also think of this as a posterior estimate, due to the sequential nature of the estimation process (see below), in that there is a distribution over 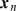 prior to the observation at a single time point 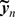. For the Gaussian approximation to this density, we have 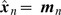 where 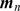 is the mean of the Gaussian.

It is also possible to improve the above estimates retrospectively
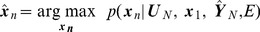
(16)where 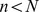. For example, upon leaving an underground metro system and turning left you may not know that you are heading north until you encounter a familiar landmark. You can then use this observation to update your estimate about where you have been previously. Estimation of 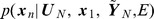 requires forward and backward inference over hidden states (see equation 30). The Gaussian approximation to this density has mean 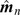, so that under the local linear approximation we have 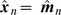.

#### Decision making

Given initial state 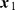, a sequence of putative motor commands 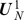 (eg. left turn), and sensory goals 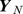, an agent can compute the likelihood of attaining those goals, 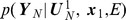. This computation requires a single sweep (or ‘replay’ - see discussion) of forward inference (see equation 29 in the section on ‘Likelihood’ below). The agent can then repeat this for a second putative motor sequence (eg. right turn), 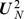, and decide which turn to take based on the likelihood ratio.
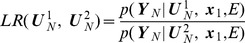
(17)Here 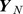 are internally generated task goals rather than sensory input from the environment 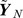. Decisions based on the likelihood ratio are statistically optimal [38]. In probabilistic models of sequential data the likelihood can be computed by a single forward pass of inference, as described below. We would therefore need two forward passes to compute the LR, one for each putative motor sequence.

This formulation of decision making is based on sets of motor primitives being combined to form actions such as ‘turn left’ or ‘turn right’. This can therefore also be regarded as motor planning (see below) at some higher level. Additionally, the generation of sensory imagery can be viewed as a component of decision making because, to evaluate the likelihood, sensory goals must be compared with sensory predictions from the agent's generative model. In later sections we consider sensory imagery in its own right.

#### Model selection

Given motor efference copy 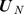, and sensory input 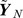 the agent computes the likelihood ratio under two different models of the environment. The agent's best estimate of which environment it is in, is given by the maximum likelihood estimate
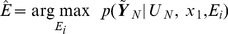
(18)For consistency with terminology in statistics, we refer to this as model selection. This can be implemented using multiple sweeps of forward inference, one for each potential environment. The likelihood can be computed, for example, for two maze models 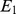 and 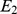 each hypothesising that the agent is in a particular environment. To decide which environment the observations are drawn from one can compute the likelihood ratio
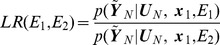
(19)where each probability is computed using equation 29 in the section on ‘Likelihood’ below.

#### Motor planning

Given current state 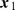 and sensory goals, 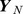, planning can be formulated as estimation of a density over actions 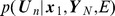, as depicted in Figure 2. This requires a forward sweep to compute the hidden states that are commensurate with the goals, and a backward sweep to compute the motor commands that will produce the required hidden state trajectory. This is described in the section below on ‘Inference over Inputs’ and can be implemented using equations 33 and 34. The agent's best estimate of the motor commands needed to attain sensory goals 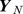 is given by the maximum likelihood estimate
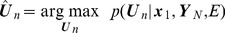
(20)Here 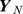 are internally generated task goals rather than sensory input from the environment 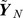.

### Forward and Backward Inference

Text S2 describes how the required probability densities can be computed at the very general level of manipulations of probability densities. However, these operations cannot be implemented exactly. They can only be implemented approximately and there are basically two types of approximate inference methods. These are based either on sampling [39] or Local Linearization (LL) [40]. In this paper we adopt an LL approach although this is not without disadvantages. We return to this important issue in the discussion. The following subsections describe the forward and backward inference algorithms under LL assumptions. Readers unfamiliar with statistical inference for dynamical systems models may benefit from textbook material [38].

#### Forward inference over hidden states

The problem of estimating the hidden states given current and previous sensory states is solved using Forward Inference. This produces the marginal densities 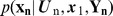. Estimation of the state 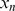 is based *only* on information up to that time point. For Linear Dynamical Systems (LDS), forward inference corresponds to the Kalman Filter, and for nonlinear dynamical systems under LL, forward inference can be instantiated using an Extended Kalman Filter (EKF) [40]. After local linearisation the state-space model can be written as
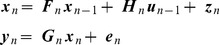
(21)where 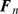, 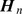 and 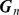 are Jacobian matrices (see Text S1 and below). There is a long history of applying KFs, EKFs and related state-space models to the problem of localisation [20], [36]. Indeed one of the key implementations of the KF is for solving the localisation problem. These probabilistic algorithms have been used in a formalism known as Simultaneous Localisation and Mapping (SLAM) [37]. The goal of SLAM research is to develop an algorithm that would allow an agent to explore and map novel environments.

In the context of localisation, forward inference allows information from path integration and sensory input to be combined in an optimal way. Under a local linear approximation the state estimates are Gaussian
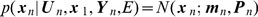
(22)and these quantities can be estimated recursively using an EKF. Here 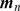 is the agent's estimate of 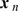 based only on information up to time index 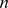. The covariance 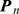 quantifies the agent's uncertainty about 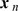, again based on information up to that time point. The agent's best estimate of location, based on forward inference, is then given by the first two entries in 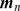 (the third and fourth entries are speed and direction, see equation 3). The EKF equations can be expressed in two steps. The first is a prediction step
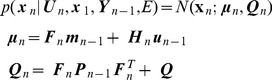
(23)where 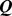 is the state noise covariance defined earlier. During localisation this corresponds to probabilistic path integration. The second is a correction step
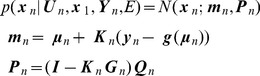
(24)where the ‘Kalman Gain’ is
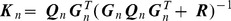
(25)and the 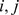
th entry in 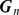 is given by
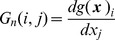
(26)evaluated at 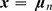. The correction step provides optimal combination of probabilistic path integration with sensory input. More specifically, probabilistic path integration produces an estimate of the current state 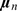. The agent produces a prediction of sensory input 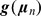 and compares it with actual sensory input 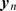. The final estimate of the current state is then 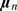 plus the Kalman gain times the prediction error 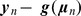. This very naturally follows predictive coding principles, as described below in the section on Neuronal Implementation. Together, the above updates implement an EKF and these recursions are initialised by specifying the initial distribution over hidden states.
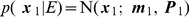
(27)


#### Likelihood

As described in Text S2, we can use the predictive densities to compute the likelihood of a data sequence. Under local linearisation the predictive density is given by
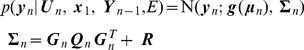
(28)The log-likelihood of a sequence of observations is then
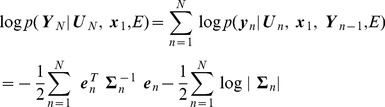
(29)where 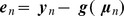 is the prediction error. The (log) likelihood of sensory input 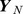 can thus be computed using equation 29. The first term in this equation corresponds to an accumulation of sum-squared prediction errors weighted by the inverse variance (precision). During decision making, the likelihood of attaining sensory goals 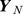 under a proposed control sequence 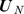 is computed using this method. During model selection, the likelihood of sensory observations 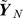, under a proposed model of the environment, 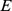, is also computed using this method.

#### Backward inference over hidden states

Forward inference over the states is used to estimate a distribution over 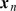 using all observations up to time point 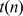. Backward inference over the states can then be used to improve these estimates by using observations up to time point 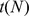 i.e. future observations. The resulting estimates are therefore retrospective. An example of when this retrospective updating is beneficial is when the observation of a new landmark disambiguates where you have previously been located. For locally linear systems, Backward Inference over states is implemented using
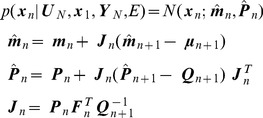
(30)Here, 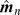 is the optimal state estimate given all sensory data up to time 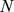. Intuitively, the state estimate based on data up to time 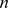, 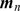, is improved upon based on state estimates at future time points (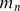 for 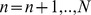). The resulting sequence 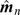 will provide more accurate state estimates than those based on purely forward inference, 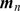.

The above formulae are known as the ‘gamma recursions’ (see Text S2). An alternative algorithm for computing 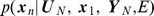, based on the ‘beta recursions’, requires storage of the data sequence 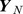 and so is not an online algorithm. The gamma recursions may therefore have a simpler neuronal implementation (see below).

The above recursions depend on a number of quantities from forward inference. These are 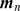, 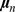, 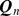 and 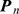. The gamma recursions are initialised with 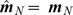 and 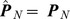. For an LDS the above equations constitute the well-known Rauch-Tung-Striebel (RTS) smoother. Various reparameterisations can be made to remove computation of matrix inverses [41]. A predictive coding interpretation is readily applied to the second row of the above equation. The backward estimate 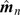 is equal to the forward estimate 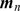 plus a correction term which is given by a learning rate matrix 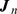 times a prediction error. This prediction error is the difference between the estimate of the next state based on the entire data sequence, 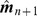, minus the prediction of the next state based only on data up to the current time point, 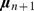.

#### Inference over inputs

This section describes forward and backward inference over hidden states and inputs. If the controls are unknown we can estimate them by computing 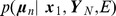 where 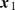 is the current state and 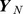 are the desired sensory states. This probability can be computed via forward and backward inference in the following locally linearised model
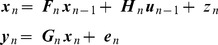
(31)with 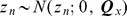, 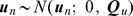 and 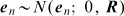. The initial control values are distributed as
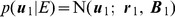
(32)Informally, the forward sweep is necessary to compute the hidden states that are commensurate with sensory goals, and the backward sweep for computing the inputs that will produce the required state trajectory. Text S3 shows how inferences about the unknown controls can be made by creating an augmented state-space model and using the previously described equations for forward and backward inference over the states. The density over estimated inputs is a Gaussian
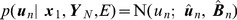
(33)with mean 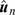 and covariance 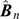. In the absence of correlations between inputs and hidden states the backward inference formulae have the simplified form
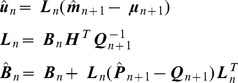
(34)


Effectively, the optimal inputs are estimated using a model-based deconvolution of the desired sensory states.

## Results

This section describes computer simulations showing how the agent's model can be used to generate visual imagery, and how inference in that model can implement decision making, model selection and motor planning. Here, ‘model selection’ refers to estimating which model of the environment is most likely given sensory data. An agent would use this to figure out what maze it was in.

In what follows we assume the agent is already equipped with the correct dynamical model 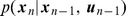. The first section below describes a preliminary learning phase in which the sensory mapping 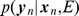 is learnt for a given environment 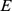. Once the agent has a dynamical and a sensory mapping it is in effect equipped with a model of its environment which can be thought of as its own virtual reality system. It can then predict the sensory consequences of the control signals it receives.

The degree to which each sensory modality is used in the following simulations is determined by the relative values of observation noise covariance (see Text S4 for details). Here we set 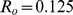, 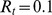 and 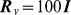 (see equation 10). This means that the agent is guided most by olfaction and touch, and least by vision. Note, however, that as there are many more visual than somatosensory or olfactory inputs this differential weighting is perhaps less distinct than it might first appear. All the simulations use 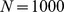 time points with a time step of 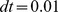. The simulations also used a very low level of dynamical noise, 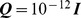, except for the planning example where we used 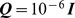.

### Sensory Imagery

This section describes a preliminary learning phase in which an agent is exposed to an environment to learn the sensory mapping from states 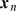 to observations 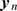. Here the agent is provided with the observations 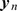 and also exact knowledge of the hidden states 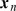. More realistic simulations would also require the agent to infer the hidden states 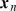 whilst learning. This is in principle straightforward but is beyond the scope of the current paper, as our focus is on temporal dynamics. We return to this point in the discussion.

The olfactory and sensorimotor models use a 10-by-10 grid of basis cells giving 100 cells in all. We assume that the parameters governing the location and width of these cells have been set in a previous learning phase. The weight vectors 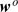 and 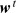 (see equations 11 and 12) were optimised using least squares regression and 225 training exemplars with uniform spatial sampling. The retinal model used the same number and location of basis cells. It additionally used 32 head direction cells each having a directional precision parameter 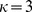. The conjunctive representation comprised 3200 basis cells. The weight vector 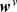 (see equation 14) was optimised using least squares and a training set comprising 10,575 exemplars. These were generated from spatial positions taken uniformly throughout the maze. Visual input from the environmental model for multiple directions at each spatial location was used to create the training examples. At the end of this learning phase the agent is exquisitely familiar with the environment.

**Figure 3:**
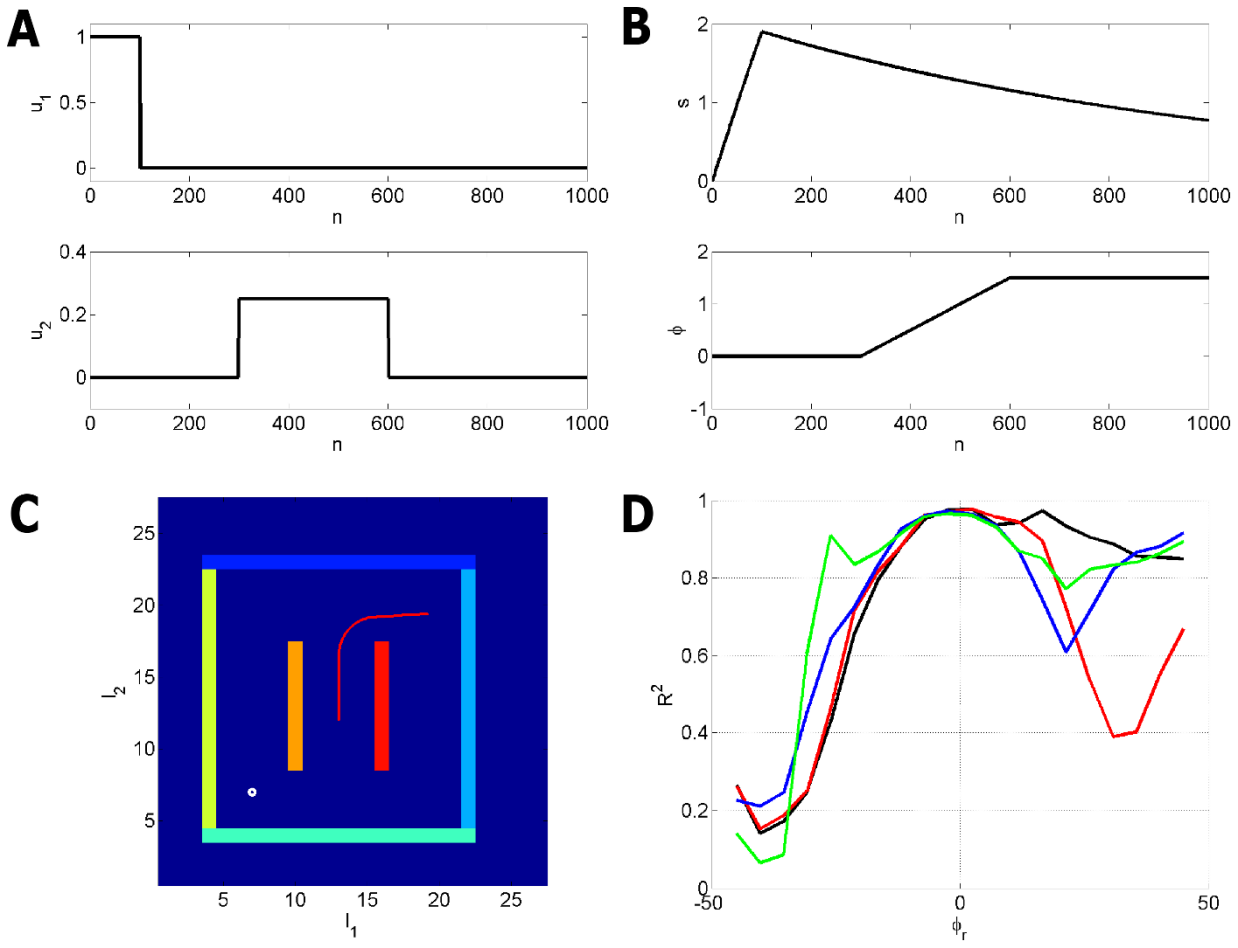
Visual imagery. (A) Control sequence used to generate visual imagery for the ‘north-east’ trajectory. The input signals are acceleration, 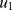, and change in direction, 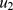. These control signals change the agent's state according to equation 3. (B) The state variables speed 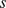 and direction 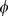 produced by the control sequence in A. (C) The state variables 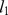 and 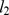 shown as a path (red curve). This is the ‘north-east’ trajectory. The state variable time series in B and C were produced by integrating the dynamics in equation 3 using the local linearisation approach of equation 5. (D) Accuracy of visual imagery produced by agent as compared to sensory input that would have been produced by the environmental model. The figure shows the proportion of variance, 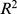, explained by the agent's model as a function of retinal angle, 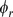. This was computed separately for the north-east (black), north-west (red), south-east (blue) and south-west (green) trajectories. Only activity in the centre of the retina is accurately predicted.

A trained model can then be used to generate visual imagery. This is implemented by specifying a synthetic control sequence, running path integration and generating predictions from the model. For example, Figure 3A shows a control sequence that is used to generate the ‘north-east’ trajectory shown in Figure 3C. We also generated ‘north-west’, ‘south-west’ and ‘south-east’ trajectories by changing the sign of direction change, 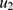, and/or the initial direction, 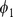.

To quantitatively assess the accuracy of these imagery sequences, 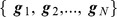, we compared them to the sequence of visual inputs that would have been received from the environmental model, 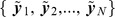. Figure 3D plots the proportion of variance explained by the agent's model as a function of retinal angle. These plots were computed separately for each trajectory, and show that only activity in the central retina is accurately predicted. This is due to the increased optic flow in peripheral regions of the agent's retina. The asymmetry in Figure 3D is due to the particular spatial arrangement and numerical values of the visual cues. These results suggest that it would be better to have a retina with lower spatial resolution in the periphery.

### Localisation

This simulation shows how an agent can localise itself in an environment. The agent was located centrally and moved according to the south-east trajectory. Its exact path was computed using noiseless path integration and the appropriate environmental inputs were provided to the agent.

In the discussion section below we propose a mapping of the forward and backward inference equations onto the hippocampal-entorhinal complex. We now report the results of two simulations. The first used the standard forward inference updates in equations 23 and 24. This corresponds to the algorithm that an agent with an intact hippocampus would use. The second, however, had a ‘lesioned hippocampus’ in that only the path integral updates in equation 23 were used (we set 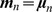). This in effect removed the top down input from hippocampus to MEC (see ‘Localisation’ subsection in the discussion) so that path integral errors are not corrected by sensory input. In both cases the agent's path updates, 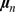, were subject to a small amount of noise (with standard deviation 0.01) at each time step.

**Figure 4:**
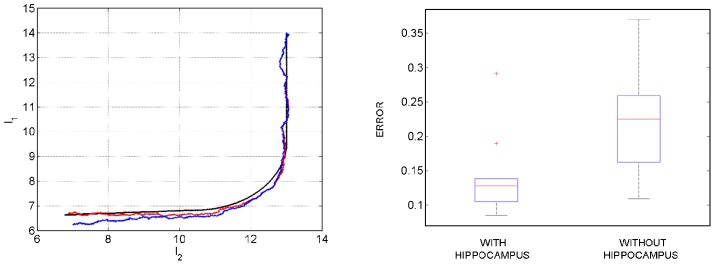
Localisation. Left: Representative result from a single trial showing true route computed using noiseless path integration (black curve), localisation with a noisy path integrator and no Hippocampus (blue curve) and localisation with a noisy path integrator and a Hippocampus (red curve). Right: Boxplots of localisation error over trials with medians indicated by red bars, box edges indicating 25th and 75th percentiles, whiskers indicating more extreme points, and outliers plotted as red crosses.

Figure 4 shows the results for single and multiple trials. Here, localisation with an intact hippocampus results in better tracking of the agent's location. Localisation accuracy was assessed over multiple trials (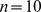) and found to be significantly more accurate with, rather than without, a hippocampus (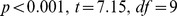). The mean localisation error was 60 per cent smaller with a hippocampus.

For the above simulations we disabled somatosensory input by setting 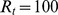. This was found to be necessary as this input is not a reliable predictor of location (the distance from a boundary is the same at very many locations in an environment).

### Decision Making

This simulation shows how an agent can make a decision about which direction to turn by computing likelihood ratios. To demonstrate this principle, we selected the ‘north-west’ and ‘north-east’ trajectories as two possible control sequences. The sensory goal 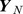 was set equal to the sensory input that would be received at the end of the ‘north-east’ trajectory. This goal was set to be identical at all time points 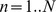.

**Figure 5:**
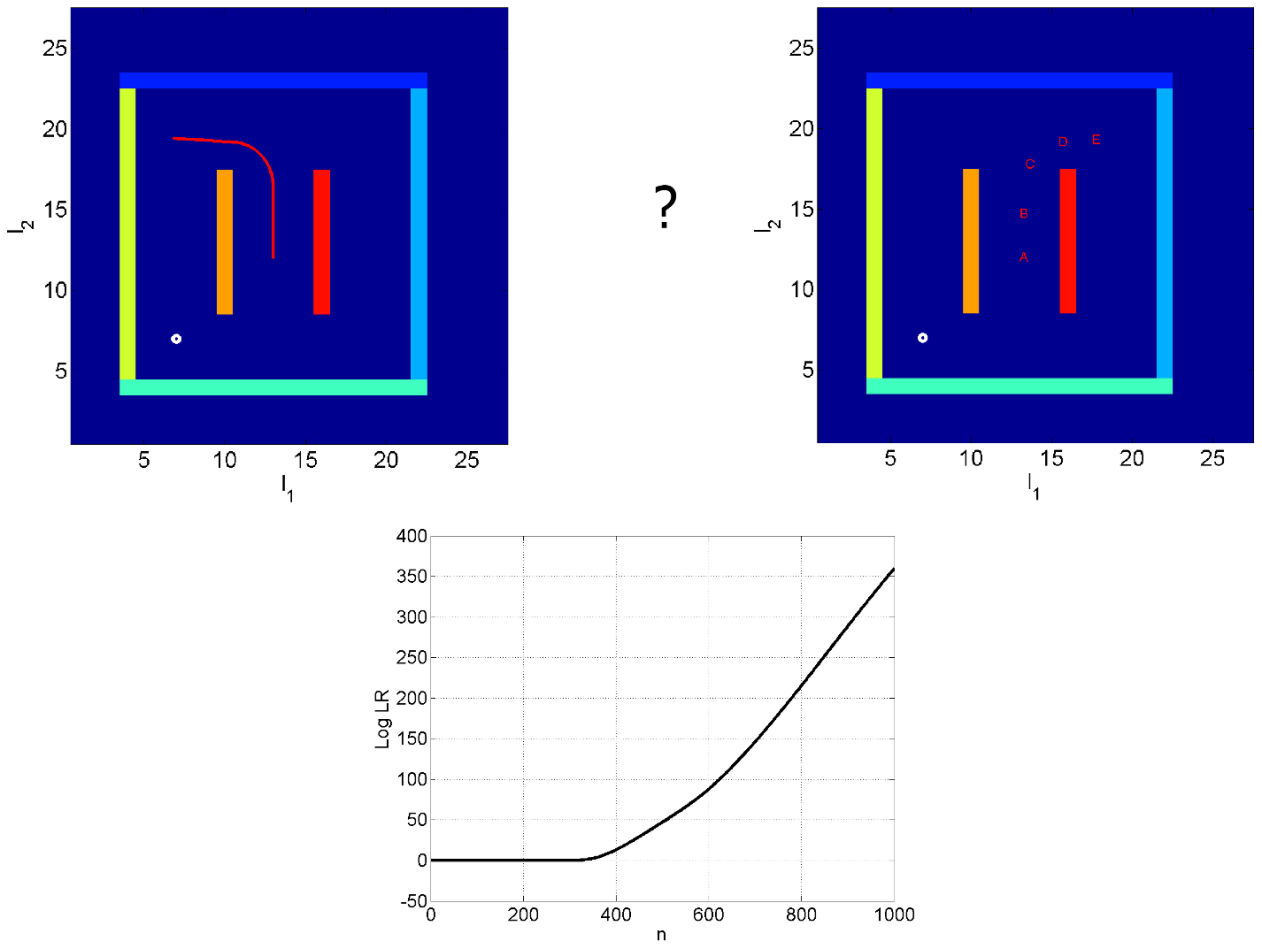
Decision making. The task of decision making is to decide whether to make a left or a right turn (hence the question mark in the above graphic). Top Left: Locations on the route of the ‘left turn’ or north-west trajectory (red curve) Top Right: The markers A, B, C, D and E denote locations on the ‘right turn’ or north-east trajectory corresponding to time points 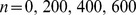 and 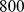 respectively. Bottom: The log likelihood ratio (of north-east versus north-west), 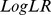, as a function of the number of time points along the trajectory.

The agent's starting location was 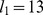 and 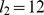 with initial speed set to zero. The log of the likelihood ratio (see equation 28), 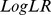, for model 1 versus model 2 was then computed at each time step. Figure 5 shows the accumulated 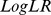 as a function of the 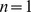 to 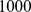 time points along the trajectory. A 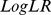 of 3 corresponds to a probability of 95% [42]. This indicates that a confident decision can be made early on in the hypothesized trajectories.

The degree to which each sensory modality is used in the above computations is determined by the relative values of observation noise covariances (see Text S4). These were initially fixed to the values described at the beginning of the simulations section. Whilst a confident decision could soon be reached using the above default values, decomposition of the LR into modality specific terms showed a strong contribution from both olfactory and visual modalities, but a somatosensory contribution that was initially rather noisy. This is due to small idiosyncrasies in the predictions of somatosensory values. We therefore experimented with the level of somatosensory noise covariance. Figure 5 was produced using a value of 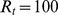 which means LR effectively ignores this contribution (although we also have 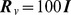, there are 20 visual inputs).

### Model Selection

This simulation shows how likelihood ratios can also be used to estimate what environment an agent is located in. We first trained an agent on the maze as described in the imagery section. We refer to this as environment one and the model, described by the set of estimated weights 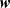, as model one. We then trained the agent on a second environment and allowed it to develop a separate model. These are referred to as environment two and model two. The second environment was exactly the same as the first except that the east and west boundary walls had their colours swapped.

**Figure 6:**
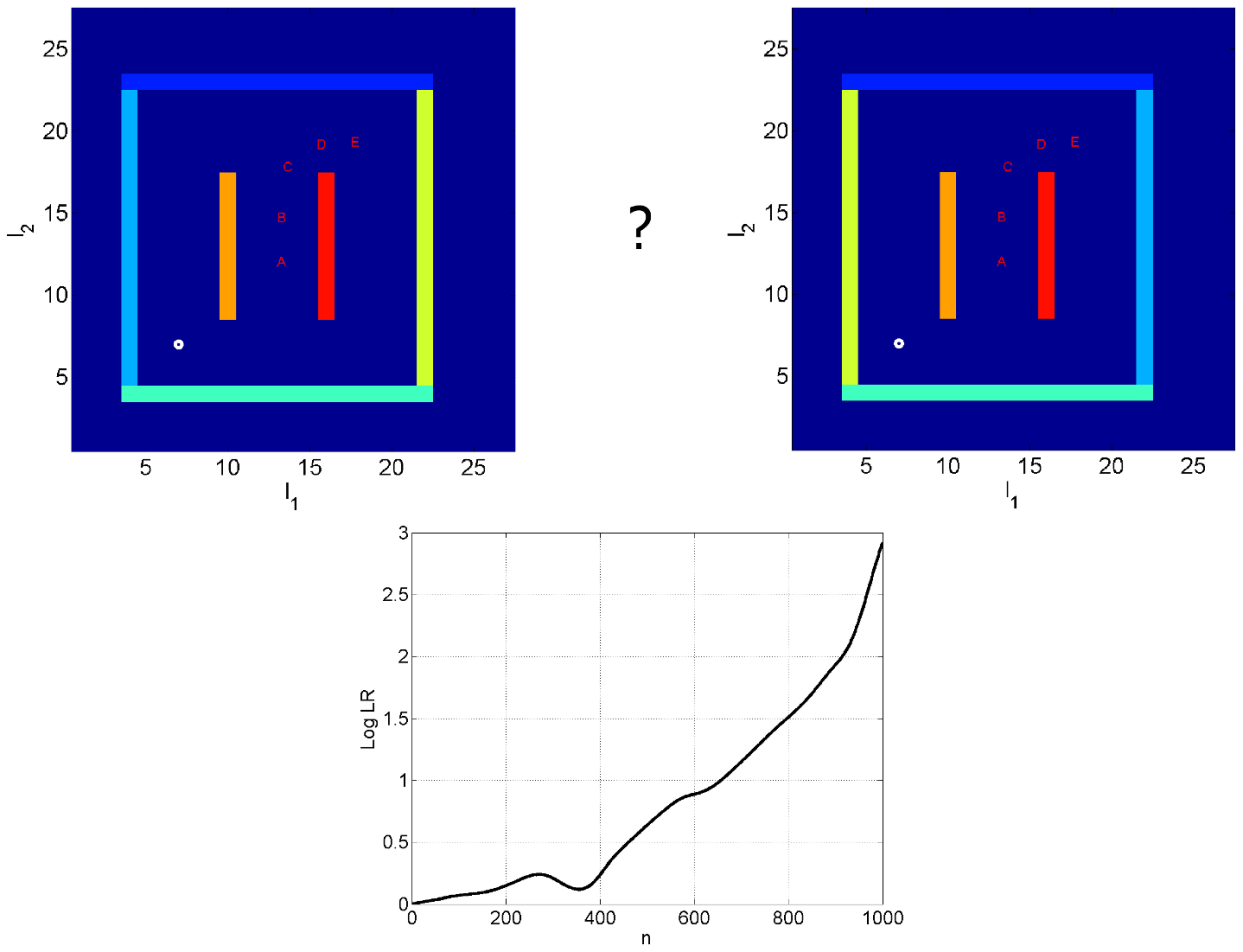
Model selection. The task of model selection is for the agent to decide which environment it is in (hence the question mark in the above graphic). Top Left: North-east trajectory in maze 2, Top Right: North-east trajectory in maze 1. The mazes have different coloured east and west walls. The markers on the trajectories (A, B, C, D and E) denote locations corresponding to different time points (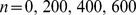 and 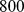). Bottom: The log likelihood ratio (of maze 1 versus maze 2), 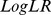, as a function of the number of time points along the trajectory. At *n* = 1000, the LogLR is approximately 3. This allows the agent to infer, with 95% probability, that it is located in maze 1 rather than maze 2.

We then placed the agent in the first maze and used the ‘north-east’ control trajectory, 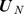, and allowed the agent to compute the likelihood of observed data under its two models, 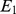 and 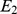, as described earlier. The log of the likelihood ratio, 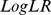 for model 1 versus model 2 was then computed at each time step. Figure 6 shows the 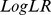 as a function of the number of time points along the trajectory.

The degree to which each sensory modality is used in the above computations is determined by the relative values of observation noise covariances. These were fixed to the values described at the beginning of the simulations section. However, because the only difference between the two models is in their predictions of retinal input (due to the swapping of wall colours), the above computation is driven solely by vision.

For the decision making example, described above, the likelihood of reaching the goal given the two trajectories is also differentiated by the olfactory inputs at the goal location (as the olfactory source is located in the south west corner and diffuses isotropically, there will be weaker input in the north east than north west corner). This explains the scaling differences in the likelihood ratios - decision making is easier, in this example, as it is guided by olfaction as well as vision. This is not generally the case, however, and only occurred here due to the specifics of the environments and goals (same olfactory sources at same locations in both mazes, different olfactory inputs at the two goals).

### Route and Motor Planning

This simulation gives an example of how route and motor planning can be implemented. The agent is placed in maze 1 at starting location 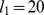, 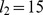 with initial speed 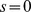 and direction 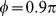. This initial state, 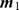, is known with high precision 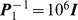 (see equation 27). The initial distribution over motor controls has mean 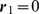 and precision 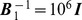 (see equation 32). The covariance of the noise on the motor controls is set to 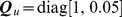 (see equation 31). This specifies that the control signals for changes in acceleration (first element) are expected to be larger than those for direction (second element). For this simulation we augmented the sensory vector 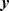 with observations of the agent's speed 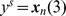.

The sensory goal 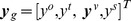 is multimodal with components for olfaction, touch, vision and speed. For olfaction, touch and speed we set 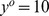, 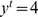 and 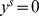. The goal is therefore to navigate to the point in space with olfactory code most similar to 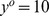. The environmental location with this value is 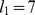, 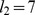. The observation noise covariance for speed was set to 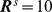. A second aim is that the distance to the nearest boundary should be close to 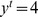. A third aim is that the speed should be as near to 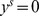 as possible. That is, the agent should be stationary at the target. The visual component 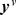 is set to correspond to an image of the left wall with all ‘yellow’ values. The desired goal trajectory, 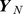, is set to be equal to the goal 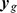 at all time points.

The degree to which each sensory modality is used in motor planning is determined by the relative values of observation noise covariance. We used the values described at the beginning of the simulations section. This means that motor planning is guided most by olfaction and touch, and least by vision. The estimated hidden states and inputs were then computed as shown in the earlier section on ‘Inference over Inputs’.

**Figure 7:**
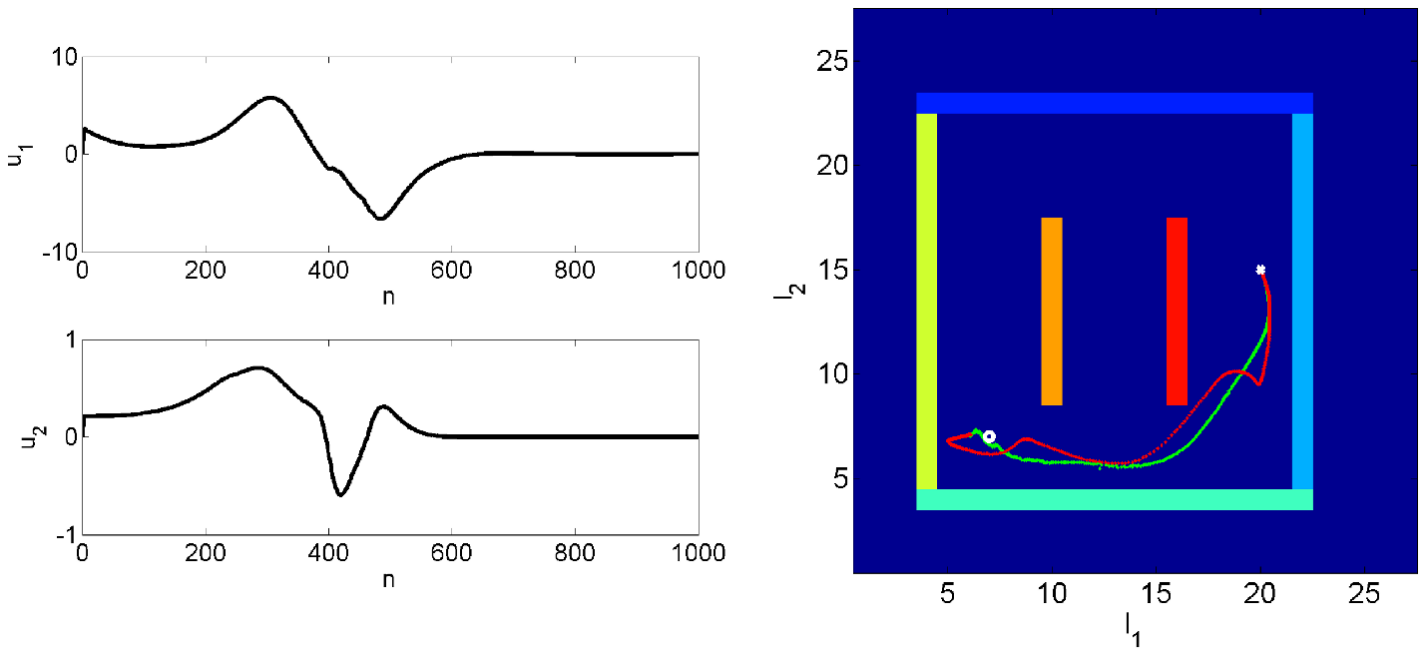
Route and motor planning. Right: The figure shows the planned route traced out by forward (red) and backward (green) inference. For forward inference we are plotting the 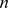 and 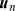 elements of 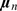, and for backward inference the 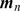 and 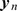 elements of 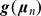. The agent is located at 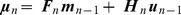 (white cross) and the goal is at 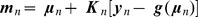 (white circle). Left: The figure shows the estimated motor control sequence for producing the desired sensory goals. This sequence corresponds to the mean from backward inference, 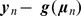, as described in the theory section on ‘Inference over Inputs’.

Figure 7 shows the planned route traced out by forward and backward inference. For forward inference we are plotting the 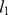 and 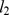 elements of 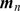 (see equation 24), and for backward inference the 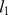 and 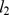 elements of 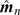 (see equation 30). The paths for backward inference are smoother and more direct. Figure 7 also shows the estimated motor control sequence. These sequences correspond to the mean from backward inference, 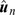, as described in the section on ‘Inference over Inputs’ (see equation 33).

Simple decisions such as ‘turn left’ or ‘turn right’ can be implemented using the ‘decision making’ procedure described in the above section. This is a rudimentary form of planning. The route and motor planning described here is a more powerful approach that we envisage is engaged when the optimal route to a goal involves the chaining together of multiple decisions (eg. ‘turn left’, ‘straight on’, ‘turn right’).

## Discussion

This paper has illustrated how the various computations underlying goal-directed spatial cognition can be implemented using statistical inference in a single probabilistic model. This extends previous work which has focussed on single computations such as localisation [20] or model selection [21]. Here we use a single model, and show that inference based on different combinations of known and unknown variables can additionally implement goal-based planning and decision making, and have shown how a specific implementation based on a continuous state space model and local linearisation can achieve these ends. In what follows we describe a neuronal implementation of our approach and discuss how the underlying forward and backward algorithms may relate to recent empirical findings of pattern replay. We close by describing a number of experimental predictions suggested by the model.

### Neuronal Implementation

**Figure 8:**
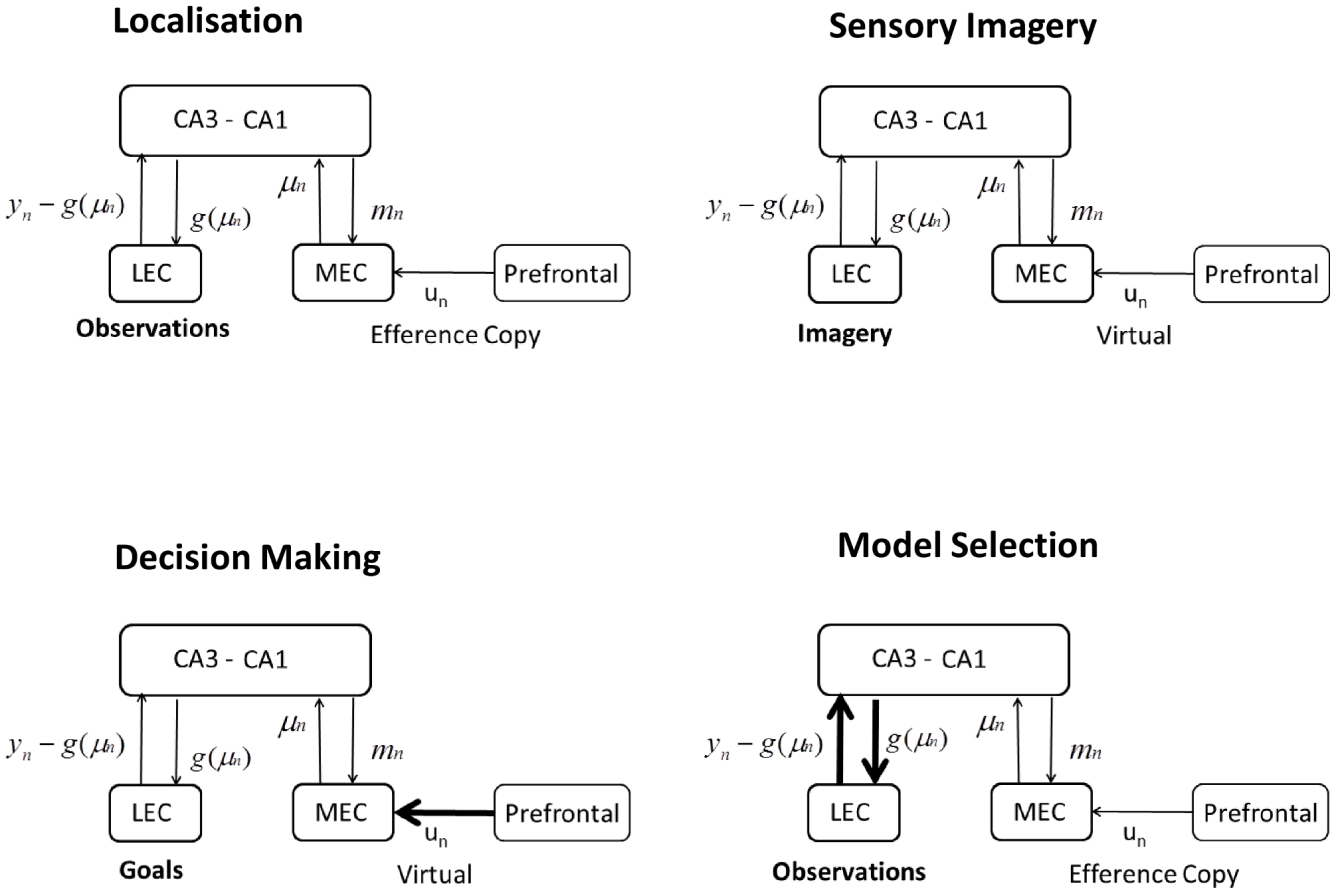
Neuronal implementation. Here 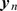 indexes time and we have control signals 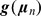, path integral hidden state estimates 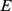, Bayesian state estimates, 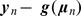, non-spatial sensory states, 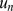 and predictions of non-spatial sensory states 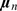. During **Localisation**, path integration in MEC combines previous state estimates and motor efference copy to produce a new state estimate, with mean 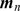 as described in equation 23. Bayesian inference in CA3-CA1 combines path integration with sensory input to get an improved state estimate 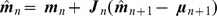 as described in equation 24. LEC sends a prediction error signal 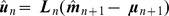 to CA3-CA1. The computations underlying ‘sensory imagery’, ‘decision making’ and ‘model selection’ are discussed in the main text in the section on ‘Neural Implementation’. CA: Cornu Ammonis, LEC/MEC: Lateral/Medial Entorhinal cortex.

This section discusses how and where in the brain the above computational processes might be implemented. Our starting point here is Figure 8 which describes a candidate set of brain regions. Entorhinal cortex is partitioned into Lateral (LEC) and Medial (MEC) components, with the latter representing spatial and the former non-spatial information [43]. The LEC receives substantial input from perirhinal cortex which in turn receives major projections from temporal cortices, whereas the MEC receives substantial input from parahippocampal cortex which in turn receives projections from parietal cortices. The anatomical connectivity supporting this architecture is described in Figure 3 of [44]. We assume that temporal, parietal, parahippocampal and perirhinal cortices and the machinery that feeds into them, together produce a compact coding of spatial and non-spatial aspects of the agent's environment. These processes are not explicitly modelled in this paper.

Our simple and tentative mapping onto hippocampal neuroanatomy currently does not distinguish between CA3 and CA1, instead we consider a single hippocampal node encompassing the activity of CA3-CA1 place cells. Our model then comprises two hippocampal-entorhinal loops, one spatial and one non-spatial, as shown in Figure 8 (top left). The spatial loop proceeds from superficial MEC layers to CA3-CA1, and returns to deep layers of MEC. This partitioning into deep and superficial layers is consistent with known anatomy and previous functional models [45]. Anatomically, entorhinal-hippocampal connectivity is more complex with, for example, direct connections from EC layer three to CA1 [46], and return connections via proximal CA1 (CA1p) and distal Subiculum (SUBd) [47], but our model does not have this level of detail.

The non-spatial loop proceeds from superficial LEC layers to CA3-CA1, and returns to deep layers of LEC. The sensory states of our spatial model, 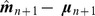, are compact codes representing non-spatial information in the superficial layers of LEC. Predictions of these sensory states from the agent's model, 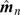, are made via the CA3-CA1 to LEC pathway. In our model, the function of CA3-CA1 is to integrate spatial input from MEC with non-spatial input from LEC. This is consistent with a recent schematic model [48], where it is argued that this functionality is preserved across mammals.

The mapping from CA3-CA1 to LEC generates the agent's predictions of sensory states, whereas the mapping from LEC to CA3-CA1 implements the (approximate) inverse of this mapping. Together, these recurrent connections constitute the agent's model of its environment, 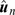, and different models will be instantiated in different subsets of these connections. That populations of cells in LEC encode sensory prediction errors, 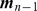, is supported by recent recordings in rats [49]. This study identified cells that fired at locations where objects had been located on previous trials (high prediction error), but did not respond when the object was actually present (no prediction error).

#### Grid, place and direction cells

Our model assumes that path integration takes place in the Entorhinal Cortex. A number of computational models of the underlying processing have appeared in the literature [45], [50], [51] and assume that allocentric space, direction and velocity are represented by populations of grid cells. These grid cells were originally discovered in rat Entorhinal Cortex (EC) and represent space using a Fourier-like basis set [52]. More recently, an fMRI study has found evidence of grid-cell-like representations in human EC [53].

Our model also assumes representations of space in CA3-CA1 which we envisage are supported by the activity of place cells. These place cells fire bursts of action potentials when a rat passes through a particular location in their environment [54]. Place cells have also been found in humans using intracranial unit recordings [55], and neuroimaging of human subjects has implicated the hippocampus in navigation [56] and the representation of spatial location [57]. A representation of spatial distance has also been identified in left hippocampus [58]. Hidden state representations of direction, in our model, are perhaps encoded by head direction cells. These neurons fire in relation to an animal's direction of heading regardless of its current location, and have been found in postsubiculum, retrosplenial cortex, anterior thalamus, striatum and entorhinal cortex [59]. Additionally, directionally modulated grid cells have been found in entorhinal cortex [60].

In summary, the speed, location and direction variables that comprise the agent's hidden state are most likely represented in a highly distributed manner in the brain, using basis representations built on cell types with multiple dependencies. In EC these will be grid cells and in CA3-CA1 these will be place cells. This level of detail is omitted from our model, as our focus is on temporal dynamics.

**Figure 9:**
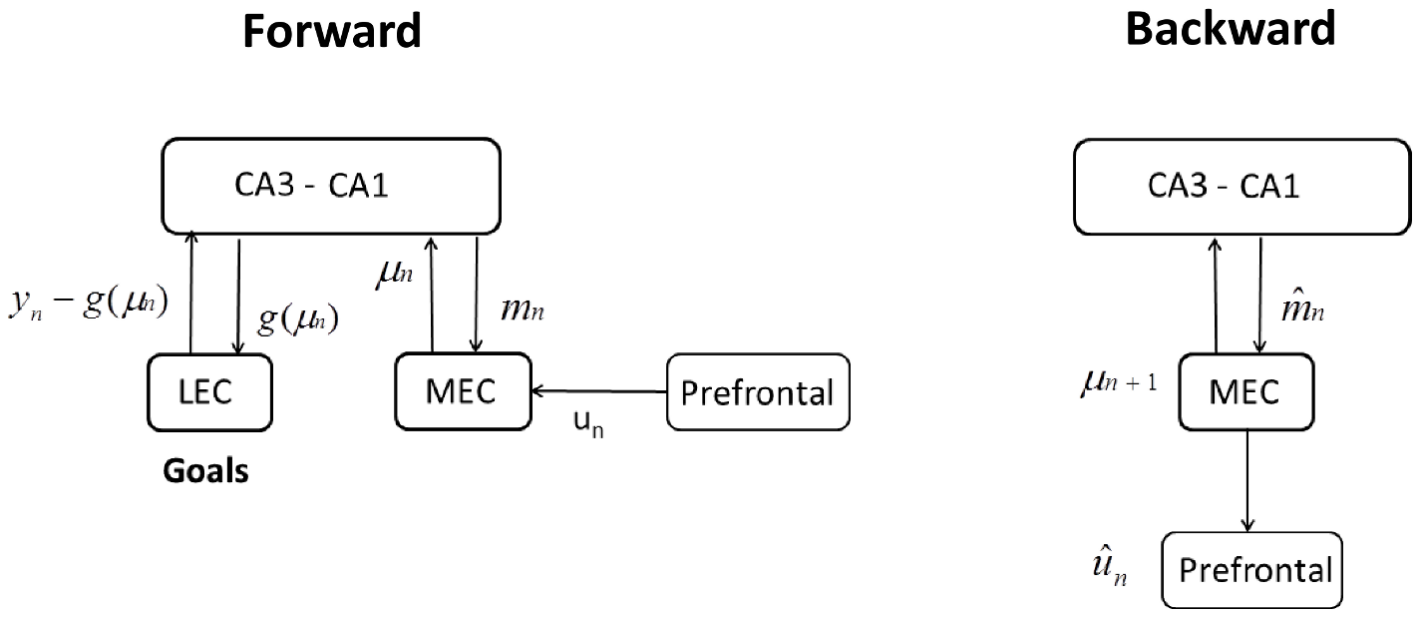
Motor and route planning. Route planning can be implemented using **Forward** inference, in which sensory goals are instantiated in LEC (or projections to it), and the recurrent circuitry produces state estimates from path integration 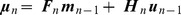, and Bayesian estimation 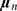, that are consistent with those goals. **Backward** inference takes as input the result of the forward sweep. It produces improved estimates of the hidden states, given by the recursion 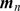, and estimates of control signals given by 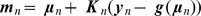. We propose that the prediction error 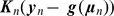 is computed in MEC and propagated to CA3-CA1 for computation of 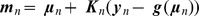 and to prefrontal regions for computation of 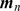. See equation 34 for more details.

Figures 8 and 9 refer to a ‘prefrontal’ module containing representations of model inputs 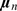 which are changes in heading direction and changes in speed. We envisage that this is a distributed circuit involving both cortical and subcortical brain regions. The subcortical regions would include for example those parts of the head direction circuit receiving proprioceptive feedback and motor efference copy [59].

#### Localisation

The architecture in Figure 8 (top left) assumes that path integration takes place in MEC, as discussed in a recent review [51]. MEC contains multi-scale grid cells which provide a basis set representation of allocentric space. In our model of spatial localisation, path integration combines previous state estimates 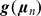 and motor efference copy 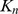 to get a new state estimate, with mean 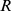 as described in equation 23.

We assume that networks in CA3-CA1 implement Bayes rule such that location estimates from path integration computed in MEC, 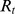, are combined with non-spatial information to form an improved estimate of location, 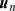. The new estimate is given by 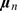 and is described more fully in equation 24. This new estimate is then fed back to MEC to be incorporated into the next iteration of path integration.

A more detailed mapping onto neuroanatomy, which is consistent with our proposal, can be motivated by concerns for how grid and place cells keep in register [61]. It has been suggested [62] that CA1 combines grid cell outputs from MEC with cue information from CA3 place cells. In our model this would correspond to CA3 computing 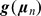 and CA1 computing 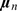. Region CA1 would then signal 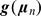 back to MEC, and the CA1 to LEC pathway could compute 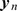 using a representation based on place cells in CA1.

The above iterative updates capture the circular nature of estimating position and direction. The activity of head direction cells [59], for example, is known to be dependent on the identification of landmarks, and on self-motion cues, such as vestibular and proprioceptive cues. Here, we envisage that vestibular cues, proprioceptive cues and self-motion contribute to probabilistic path integration and that forward inference then combines path integration with sensory input regarding landmarks. The relative contribution of path integration and sensory input, during spatial localisation, is discussed in more detail in Text S4.

The integration of sensory cues with path integral estimates of location has previously been considered in a model by Arleo and Gerstner [63]. In this model, once the error in path integration has reached a certain level the path integrator is reset using information from sensory cues. This is to be contrasted with the algorithm proposed in this paper and, for example, work by Mhatre et al. [45] in which top down predictions from CA1 to MEC continually update path integral information.

A key quantity in the combined estimate of hidden state, in equation 24, is the Kalman gain 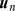. This acts as a multiplier for the prediction errors such that sensory modalities that are more predictive of hidden state have higher gain. By changing the sensory observation noise 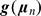 one can change elements of the Kalman gain. Indeed, our simulations on localisation showed that it was necessary to increase the somatosensory noise 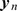 to the extent that this modality was effectively ignored during localisation (the component of the Kalman gain tended towards zero). In the brain this would be manifested by a modulation of the connection strength between somatosensory LEC and hippocampus.

#### Sensory imagery

During sensory imagery the architecture in Figure 8 (top right) is used as the agent's virtual reality engine. The MEC receives virtual motor commands, 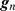, from prefrontal cortex, and uses path integration to update states, 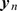. The CA3-CA1 to LEC pathway then produces predictions of sensory codes, 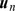. This would therefore be consistent with recent findings that the imagination of coherent scenes is hippocampus dependent [64].

The above predictions (and state estimates 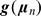) are then (separately) propagated back down cortical hierarchies, creating egocentric sensory imagery in lower-level regions of scene construction networks [65]. In the simulations described earlier, we (unrealistically) reduced these multiple stages of processing to a single mapping 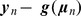.

#### Decision making

During decision making we envisage that the architecture operates as in Figure 8 (bottom left). LEC receives sensory goals, 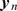, and MEC receives virtual motor commands, 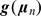, from prefrontal cortex. Sensory goals are then compared with predicted sensory input, 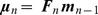 from the CA3-CA1 to LEC pathway. The likelihood of the data given the model is then proportional to the sum-squared difference between 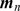 and 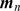 (see equation 29). Previously, Fox and Prescott [66] have proposed that septal regions, or projections to them, represent such accumulated disparities. To compute a likelihood ratio this whole process would have to happen twice, once for virtual motor commands corresponding to a left turn and once for a right turn, as described earlier. This is indicated by the thick line from prefrontal to MEC in Figure 8 (bottom left).

Experimental data [67] shows that, when rats reach decision points, potential routes are explored serially rather than in parallel, which therefore suggests that evidence for a left versus a right turn will be computed serially. To compute log-likelihood ratios it will therefore be necessary to use working memory, as in other delayed discrimination tasks. A possible neural subtrate for this are mutual inhibition circuits that can encode the alternative likelihoods [68], store them and make an appropriate decision [69].

Although we have modelled sensory goals as being represented in LEC, it may well be the case that they are represented at lower levels of cortical hierarchies. If this is the case, then the discrepancy between sensory goals and predicted sensory input would also occur at lower levels. The coarseness of these representations, and thus their anatomical instantiation, are likely to vary as a function of task requirements.

#### Model selection

During model selection we envisage that the architecture operates as in Figure 8 (bottom right). LEC receives observed sensory data, 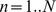, and MEC receives efference copy, 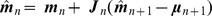, from prefrontal cortex. Sensory observations are then compared with predicted sensory input, 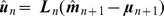, from the CA3-CA1 to LEC pathway, to produce the prediction error signal 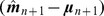. The likelihood of the data given the model is then proportional to the sum-squared prediction errors as shown in equation 29. As described above for the decision making simulations, these likelihoods may be represented in lower level sensory cortices or as accumulated discrepancy signals projecting to septal regions. Ratios of these likelihoods are used for deciding which environment an agent is in, as described above.

The recurrent connections between CA3-CA1 and LEC (thick lines in Figure 8 - bottom right) implement the agent's model of its environment. Different models will be instantiated in different subsets of these connections. To compute likelihood ratios for model selection, the above computations would have to be run twice, once for each model (we propose that this happens in parallel during ‘theta flickering’ - see below). The thick lines in Figure 8 indicate that different subsets of these connections will be engaged, corresponding to the different models.

#### Route and motor planning

During route and motor planning we envisage that the underlying neural architecture operates as described in Figure 9. This comprises separate phases of forward and backward inference. During forward inference LEC receives sensory goals, 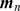, and the CA3-CA1 to LEC pathway produces predictions, 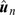. As there is no input at this stage (virtual or efference copy), MEC state estimates are driven solely by state dynamics 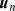 eg. location estimates are updated based on velocity and direction. The entorhinal-hippocampal loop then iteratively updates the hidden state estimates 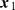, using Bayesian estimation, so as to minimise the discrepancy between sensory goals and predictions. The result is a sequence of estimates 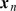 for 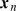 which contains a putative sequence of spatial locations that will lead to the sensory goal.

Backward inference then proceeds using just the spatial loop, as shown in Figure 8 (right panel). That sensory goals do not need to be instantiated at this stage is a consequence of using the gamma rather than the beta form of the backward recursions (see Text S2). In the absence of correlations between inputs and hidden states the update formulae for these backward recursions are straightforward, and given by equation 34. The backward estimates of the hidden states are given by the recursion 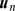 and the control signals are estimated as 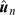. One possibility is that the prediction error 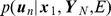 is computed in MEC and propagated to CA3-CA1 for computation of 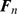 and to prefrontal regions for computation of 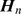, as depicted in Figure 9 (right panel). This proposed architecture is consistent with a previous suggestion that, during navigation, cue information is provided by LEC and action information by MEC [70].

### Population Codes

As with other proposals that the brain may implement some form of approximate Bayesian inference [71], to formally test this idea it is necessary to have a proposal for how neural populations represent uncertainty. Ma et al. [72], for example, have shown how populations of cells can represent probability distributions using probabilistic population codes in which simple linear combinations of firing rates can implement Bayesian inference. Beck at al. [73] have shown how such a scheme can implement Kalman filtering.

As we have locally linearised the dynamic and observation nonlinearities, the forward inference step in this paper closely corresponds to Kalman filtering. It therefore seems plausible that forward inference using EKF can be implemented using similar principles. Thus, although equations 23 to 26 perhaps seem rather removed from neurobiology there may well be a plausible neural implementation.

It has yet to be demonstrated how the gamma recursions underlying backward inference could be implemented using probabilistic population codes. However, given that the gamma recursions comprise an implementation of Bayes rule followed by a marginalisation (see Text S2) whereas Kalman filtering is a marginalisation followed by Bayes rule (see Text S2) we imagine a similar instantiation is possible.

The Beck at al. [73] approach assumes that trial-to-trial variability in population firing rates is in a class of distributions from the linear-exponential family. This includes distributions where cells have independent Poisson rates. There is good evidence to suggest that MTL cell firing is not independent and Poisson [74], but it is not known if their activity falls into the more general linear-exponential family.

Other proposals as to how the brain might implement Bayesian inference are specific to the hippocampus. One proposal [75] suggests that higher certainty is encoded by spike patterns containing more spikes and where the spikes are closer together. If this is true then our perspective makes a number of simple predictions. For example, because backward inference produces higher certainty estimates than forward inference, backward replays should produce burstier spike trains. This should be simple to test using existing data [76].

### Planning as Inference

An important part of our proposal is that the multiple tasks that together comprise spatial cognition can all be implemented using probabilistic inference in a single model. A caveat here is that our approach is restricted to goal-direction navigation. Whilst the forward inference in nonlinear dynamical systems that gives rise to the EKF algorithm, has a long history in estimates of localisation, there have been no proposals, to our knowledge, that also consider planning. However, in the machine learning literature, similar approaches for solving planning or control problems have been developed under the generic term ‘Planning as Inference’. For example, Attias [77] has proposed that planning problems can be solved using Bayesian inference.

The central idea is to infer the control signals, 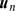, conditioned on known initial state, 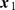 and desired goal states 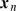. Similarly, Toussaint [78] describes the estimation of control signals using a Bayesian message passing algorithm which defaults to the classic Linear Quadratic Regulator (LQR) for linear Gaussian dynamics. Proposals have been made regarding how this Planning as Inference framework maps onto neural architectures in the brain [79], [80].

A key difference to our proposal is that Toussaint solves a closed-loop (feedback) control problem. This finds a mapping from state-space to the optimal action, also known as the ‘policy’. In terms of the underlying generative model in Figure 2, this requires extra links from 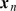 to 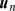. In this paper we solve an open-loop control problem. Our estimated control trajectory 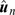 is a set of ballistic commands that cannot be updated in light of future information regarding the state of the system. Nevertheless, these commands can be rapidly computed at arbitrary time scales ‘on the fly’, and this type of control strategy may be sufficient for a compliant motor system.

### Learning

In our simulations the agent learnt to predict sensory input using a pre-developed set of place cells with fixed centres and widths. This allowed us to use a simple regression approach for learning the basis function weights, which is similar to the standard two-stage optimisation process in machine learning. In the first stage basis functions are estimated in an initial unsupervised learning phase (eg. based purely on MEC input), and basis function weights are learnt in a second, supervised learning phase [81].

Our simulations also assumed the agent had exact knowledge of its hidden state during learning, whereas more realistic simulations would also require the agent to infer these states. In principle this requires a straightforward implementation of the Expectation-Maximisation (EM) algorithm [38], [82] for learning in dynamical systems.

A more powerful alternative which integrates out the dependence on model parameters in the forward and backward passes is Variational Bayes (VB) [83], [84]. Implementation of these VB schemes would mean that the maximum likelihood approach described in this paper would be replaced by a maximum evidence approach. Agents would implement decision making, model selection and motor planning by maximising the model evidence. Given that VB approximates the model evidence using free energy, the resulting scheme would then be broadly consistent with the Free Energy Principle [85]. A further detail here is that in previous applications of VB [83], [84], backward inference was implemented using the beta not the gamma recursions. In this paper we propose that it is the gamma recursions that are implemented in the brain, as they do not require storage of sensory observation sequences.

### Local Linearisation

The forward and backward algorithms are general purpose computations which may be implemented in a number of ways and this paper has focussed on an implementation based on local linearisation. The benefit of this is that the state probability distributions are Gaussian and so may be described with a small number of parameters; means and covariances. Additionally, there are analytic formulae for updating the parameters.

A drawback of the LL approach is that the true probability distributions may be non-Gaussian. One possibility is that the distribution over the agent's location may be multimodal. This will be the case when an agent is placed in a familiar environment at an unknown location where there are multiple locations consistent with sensory data. For this scenario inferential methods based on sampling, such as particle filtering, would be more appropriate [37].

A second concern is that a single iteration of forward and backward inference may not be sufficient to find the controls that maximise the planning likelihood 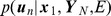. It may be possible to improve the estimated controls by running multiple forward and backward replays such that the linearisation takes place around a different and improved trajectory each time. This iterated local linearisation would be analogous to the iterative Local Quadratic Gaussian (iLQG) approach from control theory [86].

This second concern may also be addressed by treating space as discrete rather than continuous. In this perspective the agent is currently located in one of a finite number of ‘bins’ each of which may correspond to the support of a place cell. The optimal trajectory through these bins can then be computed by solving a discrete Bellman equation. Todorov has shown that this corresponds to backward inference in a hidden Markov model [87]. This computation relies on a recursive high-dimensional update that is perhaps readily suited to the massively recurrent nature of CA3. These computations would be consistent with earlier proposals that the hippocampus itself is suited for solving shortest path problems [88].

### Open-Loop Control

In regard to motor planning, this paper has described a forward and backward inference procedure which allows an agent to solve an open-loop control problem. This produces a control trajectory that is a set of ballistic commands that cannot be updated in light of future information regarding the state of the system. It is possible to augment the generative model to include extra links from states to actions, so that the agent instead learns a policy - a mapping from states to actions, as in [78]. This would then provide a solution to the closed-loop (feedback) control problem.

However, it may be the case that the mammalian brain solves the closed-loop problem in two stages. First, the computational power of recurrent networks in CA3 could be used to implement forward and backward inference to solve the open-loop problem. Estimated trajectories would then be replayed to ventral striatum during quiet wakefulness or slow wave sleep. This is consistent with an earlier model [89] and the observation of ripple activity propagating to this region [90]. These replays would then be used to train up a habitual dorsal striatal decision making system (see [11] for a review of habitual versus flexible/deliberative systems and their anatomy).

This is also consistent with proposals that for known environments, navigational control is gradually transferred from a flexible inferential system to a habitual system based on a hippocampo-striatal mapping [14]. Such a hippocampo-striatal model has previously been proposed by Foster et al. [29].

### Cognitive Control

This paper has described how the various aspects of spatial cognition can be implemented using inference in a statistical model. It has not, however, addressed the broader cognitive control issues such as how internally generated goals are produced or when to switch between localisation versus model selection versus decision making modes. A recent computational framework [22], called Information Foraging (IF), however, does address some of these issues. This approach requires that agents compute the information that will be gained by making spatial decisions, which in turn requires the agent to have a probabilistic model of its environment. Thus, it would be possible for both IF and the Forward-Backward (FB) model to both use the same underlying probabilistic model, with perhaps IF deciding when to run an iteration of FB.

This paper has proposed how model-based control may be implemented using spatial models implemented in hippocampal circuits. But it has not addressed how the control of decision making is arbitrated between, for example, model-based and model-free controllers. An influential proposal here [15] is that such arbitration is based on the confidence with which each system can make a decision. Thus, model-based and model-free systems can be combined by weighting each decision with their relative confidence. The ‘Mixed Instrumental Controller’ [19] also makes use of both types of decision making system. The model-based system incurs a fixed computational penalty reflecting the fact that model-based decisions require time to reach. If the estimated benefit of a model-based decision does not exceed this penalty then control is given to the model-free controller.

### Theta Sequences and Pattern Replay

The next and final section of this discussion summarises the specific predictions of the model proposed in this paper. To put these predictions in context we now briefly review two sets of empirical findings. These are, firstly, the observations of ‘theta sequences’ [91] which are sequential patterns of place cell firing occurring whilst rats move about in their environment and theta activity is recorded in hippocampus. The second set of observations are, again, sequential patterns of place cell firing but now occurring during sleep or quiet wakefulness and when Sharp Wave Ripples (SWRs) (henceforth ‘ripples’) [24] are recorded in hippocampus.

The phenomenon of phase precession refers to the observation [92], [93] that place cells fire at gradually earlier phases of the hippocampal theta rhythm as rats move through their place fields. This is consistent with the notion of ‘theta sequences’ in which place cells fire in sequence within a theta cycle. Theta sequences have since been measured across cell-populations [91]. Additionally, theta sequences which sweep forward in advance of a rat's current location have been observed and are especially noteworthy at decision points in maze navigation. For example Johnson and Redish [67] recorded the activity of neural ensembles in the dorsal hippocampal CA3 region of awake behaving rats running in a T-maze. They found that as rats reached a decision point, representations swept predominantly forward from the current location, first down the right path and then the left. This activity did not occur in both forward directions simultaneously: the representation first encoded one arm and then the other. Finally, Gupta et al. [4] have shown that theta sequences represent distances further ahead of a rat during acceleration and further behind during deceleration, and that these sequences represent the environment in ‘chunks’. A key feature of theta sequences is that they are time-compressed, occurring at about 5 to 10 times the speed of actual behaviour [91], [93], [94]. That is, were a rat to run through an environment at a typical speed, it could activate the same sequence of place cells, but would do so 5 to 10 times more slowly.

We now turn to the discussion of ripple activity. In humans, episodic memories are thought to be encoded by the Medial Temporal Lobe (MTL) memory system. Information regarding these memories can then be transferred to neocortex [95]–[97] and a proposed mechanism of this transfer is the replay of episodes during later waking or sleep [27] so that neocortical synaptic plasticity can then act to strengthen cortico-cortical connections. This replay activity has been observed primarily in rodents using spatial navigation tasks [98] during ripples in Slow Wave Sleep (SWS) [99] and quiet wakefulness. There is evidence that this pattern replay is related to consolidation and transfer, as disrupting ripples impairs performance in a spatial memory task [100].

Place cell sequences observed during awake ripples have been observed to be played backwards. This is known as reverse replay. Foster and Wilson [76], for example, recorded from cell ensembles in dorsal CA1 hippocampus in awake behaving rats and detected reverse replays after a rat had run the length of a 1D track. Similar reverse replays that start immediately after navigation have been observed on other 1D tracks [101], a linear path through a 2D environment [102], a 2D open-field environment [103], and a two choice T-maze [104]. Place cell sequences observed during awake ripples have also been observed to be played forwards [101]. This is known as forward replay.

Replay activity during ripples is also time-compressed, with sequences being replayed within the duration of a single ripple (50–250 ms). This corresponds to a compression factor of about 15 to 20 relative to the original behaviour [102].

The above forward and backward replays are also known as ‘local replays’ or ‘locally initiated replays’ so as to distinguish them from another phenomenon known as ‘remote replay’ or ‘remotely initiated replay’. This occurs when a rat replays an experience of one place whilst being physically located in another. In one experiment [105], rats were exposed to two different environments which had the same physical structure (allocentric layout) but differed in their set of visual cues. Replays of trajectories in one maze were observed whilst the rat was located in the other. Remote replay has also been observed [102], [104] where rats replayed activity corresponding to remote parts of the same environment. As is the case with local replays, remote replays can be forward or backward in time [104]. In general, replay activity during ripples can be forward or backward, whereas theta sequences are always forward.

Jadhav et al. [106] have interrupted awake ripples during performance of a navigation task with alternating goals in a W-shaped maze. Ripple disruption was found to affect decision making on the outbound leg of the task, which required linking of past information with current location. However, it did not affect the inbound leg which required no such memory component therefore providing evidence that awake ripples support spatial working memory.

Finally, Dragoi and Tonegawa [107] have observed ‘preplay’ activity. Here, the sequence of place-cell firing during a novel spatial experience occurred on a significant number of occasions during the resting or sleeping period prior to that experience. They propose that this activity organises hippocampal assemblies into dynamical structures ready for subsequent associations with sensory episodes.

### Model predictions

This section summarizes the predictions of our model (the ‘FB model’). We indicate where these predictions are unique to the proposed model and where they are shared by others.

#### The hippocampus optimally combines sensory cues with path integration

This prediction is not unique to the FB model. It is shared for example by the conception of the Hippocampus as a Kalman Filter [20]. Evidence for the related hypothesis that humans optimally combine sensory cues with path integration is provided in a behavioural study [108]. Given behaviorial data on a rat navigating in a simple environment in darkness and then in light, it should be possible to develop a spatial model (mapping location to sensory cues) and then infer the precision of sensory cues with respect to path integral input (ie. how much noisier one is than the other). The principles of such an investigation are the same as for the study of Bayesian sensory integration in other domains eg. visual and haptic (for a review, see [71]).

#### Local changes to an environment will produce hippocampal prediction errors

Local changes to an environment, such as objects being moved or disappearing, will be reflected in greater ‘prediction error’ activity in layer 2 LEC cells. This observation has in fact already been made in the reported activity of ‘trace cells’ in LEC [49]. This prediction is not unique to the FB model, however. It is common to all predictive coding models which posit that connections from hippocampus to LEC layer 5 convey predictions, and connections from LEC layer 2 convey prediction errors [23]. The model in Mhatre et al. [45] also has this structure, although only predictions of medial rather than lateral EC are considered. These predictive coding models can be traced back to earlier formulations by Gray and McNaughton [109] (p. 243).

#### Theta sequences during decision making are driven by prefrontal circuits

The FB model predicts that theta sequences during decision making (a la Johnson-Redish [67]) are driven by activity in prefrontal circuits. Moreover, different populations of neurons will be engaged during left-turn versus right-turn theta sequences. This prediction could be confirmed using cell assembly recordings of prefrontal cortex in rat, or using pattern recognition methods for decoding neuroimaging data in human. This prediction is similar to an earlier proposal [32] that suggested prefrontal regions signal virtual motor efference copy to a spatial cognition system during sensory imagery.

#### Different populations of CA3/CA1 cells will become active during model selection

It has long been proposed that different environments are encoded using different populations of CA3/CA1 cells. Thus, during model selection, when an agent is trying to figure out which environment it is in, we envisage that these different populations will become active as they compete to explain sensory observations. This has been observed in a recent study by Jezek et al. [110] who familiarized a rat with two different environments, which had identical allocentric layouts but different sensory cues (wall markings). They were then able to electronically switch the sensory cues. Immediately following these switches, two different populations of CA3 cells flickered on and off until one representation became stable. This is referred to as ‘theta flickering’. The FB perspective on theta flickering is as follows. By using the models developed in the investigation of sensory cue integration (see above), it should be possible to predict how long the flickering period endures. The end of the flickering period will correspond to an above threshold likelihood ratio (see Figure 5). This prediction is not unique to the FB model but would be common to any dynamic Bayesian model of hippocampal activity, such as Kalman or particle filtering [20], [111].

#### Remote replays are algorithmic and support route and motor planning

The replays observed during ripples are often considered to be of previously experienced sequences from episodic memory. We refer to this as the ‘episodic’ view. In contrast, the FB model predicts that replays are not merely previous experiences played forwards or backwards but are the result of computations (the forward and backward recursions). This perspective, which we might term ‘algorithmic’ rather than ‘episodic’ makes a number of specific predictions.

1. Because the function of remote replay is hypothesised to be planning of spatial and motor trajectories then the interruption of remote replay should result in poorer subsequent navigation performance (speed,accuracy). This prediction is specific to the FB model.
2. Backward replays should be similar but not identical to time-reversed forward replays. This is illustrated in Figure 7. More specifically, the backward replays are more direct than the corresponding forward replays. That is, they describe shorter trajectories from beginning to end. This prediction is specific to the FB model.
3. The FB model predicts that reverse replays encode location with higher spatial precision than the corresponding forward sequences. Here, decoded locations are computed in a backward replay, and FB predicts that the associated spatial precisions will be higher than for the corresponding forward replay. If spatial precision is reflected in higher density spike trains [75] then reverse replays should contain higher density spike trains than the associated forward replay. To our knowledge this prediction is unique to the FB model.
4. Forward and backward replays should be paired in that a backward replay starts from the end point of a forward replay. The backward replays must therefore be initiated immediately after completion of the corresponding forward replay. This ‘temporal pairing’ is a key prediction of the FB model but has so far not been reported in the literature.

The pairing of forward and backward replays, referred to above, would be evident when the following conditions are satisfied (i) the agent is familiar with the environment, (ii) the optimal route requires a chaining together of decisions, rather than a single decision. This is illustrated for example in Figure 7 which depicts route and motor planning. Given that the agent is initially facing south, two decisions have to be made to reach the goal (turn right and continue, rather than eg. turn right then right again). This is to be contrasted, for example, with ‘decision making’ in Figure 5, where a single decision is required to reach the goal. The agent needs to be familiar with the environment for it to have developed a model and planning is then based on this model. The above conditions would be satisfied following minor reconfigurations of a familiar environment, such as blockage of a familiar route [112] or appearance of a shortcut [113]. Having updated its model of the environment, an agent could then use forward and backward replays to plan a new optimal route to goal.

A plausible alternative functional role for remote replay is that it is involved in maintaining a memory representation of paths that have not recently been experienced [5], [104]. For example, reverse replay might provide a mechanism for developing a navigationally complete representation of an environment - one reflecting not only trajectories experienced, but also the corresponding reverse trajectories. There is also evidence, referred to earlier, that replays during awake ripples are involved in spatial working memory [106].

Just as we predict that backward replays will be more direct than preceding forward replays, we also predict that later forward replays will be more direct than preceding forward replays. This is, however, predicated on forward and backward replays being repeated iteratively (see ‘Local Linearisation’ above) and being a signature of route planning. Later forward replays can then become quite different to earlier forward replays and correspond to much more direct paths. This prediction is consistent with recent findings [104] where novel shortcut trajectories were constructed during replay activity. It is also more generally consistent with recent research [114] that replay activity is involved in planning and is a predictor of subsequent behaviour.

#### Changes in effective connectivity

We now describe predictions of the FB model that posit a change in effective connectivity from one brain region to another. In humans this can be assessed using functional neuroimaging and measures of effective connectivity [115], [116]. These human neuroimaging experiments would use previously developed virtual reality environments. Additionally, it is becoming easier to make simultaneous electrophysiological recordings from multiple brain regions in rats. To our knowledge the following predictions are unique to the FB model.

The FB model predicts that theta sequences during decision making (a la Johnson-Redish [67]) are driven by populations of neurons in prefrontal circuits. We would therefore expect to see increased effective connectivity from prefrontal to hippocampal regions at decision points. The FB model predicts that task goals during decision making are instantiated by increased connectivity from PFC to LEC. We would therefore also expect an increase in effective connectivity from PFC to LEC during these decisions. Additionally, which way to turn would be based on the computation of a likelihood ratio, which we hypothesise will employ the same PFC machinery as for other delayed discrimination tasks (see earlier section on decision making). We would therefore expect to see increased effective connectivity from hippocampus to PFC during decisions. The above predictions are consistent with recent findings of changes in theta coherence in hippocampal-prefrontal networks [117].

During sensory imagery (and decision making) we expect greater prefrontal to hippocampal connectivity, as virtual efference copy is proposed to drive activity in hippocampus. This proposal has also been made in a previous model of spatial memory and imagery [32]. During route and motor planning we expect prefrontal to LEC connectivity to be increased so as to instantiate task goals (same as for decision making above). Additionally, we expect MEC to prefrontal connectivity to be increased so that control signals can be estimated from the computed reverse path.

### Conclusion

We have shown that the various computations underlying spatial cognition can be implemented using statistical inference in a single probabilistic model. Inference is implemented using a common set of ‘lower-level’ computations involving forward and backward inference over time. We have proposed a mapping of the above computational processes onto lateral and medial entorhinal cortex and hippocampal regions CA3-CA1. This proposed mapping is consistent with recent findings in rat electrophysiology, and other proposals that one function of the hippocampus that is preserved across mammalian species, is that it integrates spatial and non-spatial information. We have also proposed that these computations are reflected in recent findings of pattern replay in the mammalian brain. Specifically, that theta sequences reflect decision making, theta flickering reflects model selection, and remote replay reflects route and motor planning. Many of the underlying hypotheses can be tested using existing data.

## Supporting Information

Text S1Contains a description of how to to derive the flow matrices 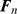 and 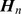 using local linearisation of state dynamics originally described using a nonlinear differential equation. This used a ‘local regression’ approach described in [28].(PDF)

Text S2 Contains a description of the general formulation of forward and backward inference for state space models, at the level of manipulations of probability densities. It describes two alternative formulations based on (i) the gamma and (ii) the beta recursions [38], [118], [119].
(PDF)

Text S3 Shows how inference over inputs can be accommodated in the standard state-space framework by using an augmented model where the hidden states are an augmented vector comprising both the original states and the inputs.
(PDF)

Text S4 Presents an alternative formulation of equation 24 in the main text, showing how the relative contribution of path integration and sensory cues, to the estimation of hidden states, is a function of their relative precision [59], [120].
(PDF)
