# Supplementary material for: Forward and Backward Inference in Spatial Cognition
Source: PLoS Comput Biol. 2013 Dec 12;9(12):e1003383. doi: 10.1371/journal.pcbi.1003383 (PMC3861045; doi:10.1371/journal.pcbi.1003383)
Supplement: Text S1 — Contains a description of how to to derive the flow matrices and using local linearisation of state dynamics originally described using a nonlinear differential equation. This used a ‘local regression’ approach described in [28]. (PDF) [file pcbi.1003383.s001.pdf]

Text S1: Forward and Backward Inference in Spatial Cognition

Will D. Penny, Peter Zeidman and Neil Burgess

Local Linearisation

If the deterministic part of the dynamics evolve according to a linear differential equation

$$\dot{\boldsymbol{x}} = \boldsymbol{A}\boldsymbol{x} + \boldsymbol{B}\boldsymbol{u} \tag{1}$$

then a discrete time update is given by

$$\boldsymbol{x}(t) = \exp(\boldsymbol{A}t)\boldsymbol{x}(0) + \int_0^t \exp(\boldsymbol{A}(t-\tau))\boldsymbol{B}u(\tau)d\tau \tag{2}$$

For time step n, if we assume that $u(t) = 0$ except at $t = t(n)$ then we have

$$\boldsymbol{x}_n = \exp(\boldsymbol{A}dt)x_{n-1} + \boldsymbol{B}u_n \tag{3}$$

where dt is the time step. If $\boldsymbol{u}_n$ is not changing quickly we have $\boldsymbol{u}_n = \boldsymbol{u}_{n-1}$. For nonlinear dynamics

$$\dot{\boldsymbol{x}} = \boldsymbol{f}(\boldsymbol{x}, \boldsymbol{u}) \tag{4}$$

then we can write

$$\boldsymbol{x}_n = \boldsymbol{F}_n\boldsymbol{x}_{n-1} + \boldsymbol{H}_n\boldsymbol{u}_{n-1} \tag{5}$$

where the flow matrices are given by

$$\begin{aligned} \boldsymbol{F}_n &= \exp\left(\boldsymbol{J}(\boldsymbol{f}, \boldsymbol{x})dt\right) \\ \boldsymbol{H}_n &= \boldsymbol{J}(\boldsymbol{f}, \boldsymbol{v})dt \end{aligned} \tag{6}$$

and $\boldsymbol{J}(\boldsymbol{f}, \boldsymbol{x})$ is the Jacobian matrix of the function $\boldsymbol{f}$ with respect to $\boldsymbol{x}$ (matrix of first derivatives). In forward inference, these are evaluated at $\boldsymbol{x} = \boldsymbol{m}_{n-1}$ and $\boldsymbol{u} = \boldsymbol{u}_{n-1}$ (for known causes) or $\boldsymbol{u} = \boldsymbol{r}_{n-1}$ (for estimated causes).

However, our evaluations of the above approximations for $\boldsymbol{F}_n$ and $\boldsymbol{H}_n$ showed considerable inaccuracies for a range of angles, ϕ. We therefore adopted the following 'local regression' approach which is similar to that proposed by Schaal [28]. This used multiple, typically 10, expansion points sampled from the previous posterior ($\boldsymbol{m}_{n-1}$, $\boldsymbol{P}_{n-1}$). For each, we evaluated the gradient $\boldsymbol{f}(\boldsymbol{x}, \boldsymbol{u})$ and estimated the next state based on a first order Euler method. We then regressed the next states onto previous states and computed $\boldsymbol{F}_n$ and $\boldsymbol{H}_n$ using least squares regression.
