# Supplementary material for: Forward and Backward Inference in Spatial Cognition
Source: PLoS Comput Biol. 2013 Dec 12;9(12):e1003383. doi: 10.1371/journal.pcbi.1003383 (PMC3861045; doi:10.1371/journal.pcbi.1003383)
Supplement: Text S2 — Contains a description of the general formulation of forward and backward inference for state space models, at the level of manipulations of probability densities. It describes two alternative formulations based on (i) the gamma and (ii) the beta recursions [38], [118], [119]. (PDF) [file pcbi.1003383.s002.pdf]

Text S2: Forward and Backward Inference in Spatial Cognition

Will D. Penny, Peter Zeidman and Neil Burgess

Forward and Backward Inference: General Formulation

In this section, for brevity we drop the dependence on $\boldsymbol{u}_n$ and E in our notation, but this is nevertheless implied. Probabilistic path integration can be implemented by computing

$$p(\boldsymbol{x}_n|\boldsymbol{Y}_{n-1}) = \int p(\boldsymbol{x}_n|\boldsymbol{x}_{n-1})p(\boldsymbol{x}_{n-1}|\boldsymbol{Y}_{n-1})d\boldsymbol{x}_{n-1} \quad (1)$$

To optimally combine path integration with sensory input we compute

$$p(\boldsymbol{x}_n|\boldsymbol{Y}_n) = \frac{p(\boldsymbol{y}_n|\boldsymbol{x}_n)p(\boldsymbol{x}_n|\boldsymbol{Y}_{n-1})}{p(\boldsymbol{y}_n|\boldsymbol{Y}_{n-1})} \quad (2)$$

which is Bayes rule with prior $p(\boldsymbol{x}_n|\boldsymbol{Y}_{n-1})$ from probabilistic path integration, likelihood $p(\boldsymbol{y}_n|\boldsymbol{x}_n)$ from sensory input, and the normalisation term is given by

$$p(\boldsymbol{y}_n|\boldsymbol{Y}_{n-1}) = \int p(\boldsymbol{y}_n|\boldsymbol{x}_n)p(\boldsymbol{x}_n|\boldsymbol{Y}_{n-1})d\boldsymbol{x}_n \quad (3)$$

which is also known as the predictive density. Equations 1 and 2 together update $p(\boldsymbol{x}_{n-1}|\boldsymbol{Y}_{n-1})$ to $p(\boldsymbol{x}_n|\boldsymbol{Y}_n)$. This procedure is referred to as forward inference and is also known as filtering [38]. The goals of forward inference are to (i) produce the 'filtering density' $p(\boldsymbol{x}_n|\boldsymbol{Y}_n)$ and (ii) compute the likelihood $p(\boldsymbol{Y}_n)$. The likelihood of a data sequence can be computed using the product of predictive densities

$$p(\boldsymbol{Y}_n) = p(\boldsymbol{y}_1)\prod_{n=2}^{N} p(\boldsymbol{y}_n|\boldsymbol{Y}_{n-1}) \quad (4)$$

If forward inference runs up until time index N then it is possible to run a backward inference procedure which produces the posterior density $p(\boldsymbol{x}_n|\boldsymbol{Y}_N)$. This is the probability of the state $\boldsymbol{x}_n$ given observations up to time index N. This includes observations after time index n and so corresponds to a retrospective update. This can be computed recursively using

$$p(\boldsymbol{x}_n|\boldsymbol{Y}_N) = \int p(\boldsymbol{x}_n|\boldsymbol{x}_{n+1},\boldsymbol{Y}_n)p(\boldsymbol{x}_{n+1}|\boldsymbol{Y}_N)d\boldsymbol{x}_{n+1} \quad (5)$$

The first term in the integral can be thought of as a reverse flow term and is computed using Bayes rule

$$p(\boldsymbol{x}_n|\boldsymbol{x}_{n+1},\boldsymbol{Y}_n) = \frac{p(\boldsymbol{x}_{n+1}|\boldsymbol{x}_n,\boldsymbol{Y}_n)p(\boldsymbol{x}_n|\boldsymbol{Y}_n)}{\int p(\boldsymbol{x}_{n+1}|\boldsymbol{x}_n,\boldsymbol{Y}_n)p(\boldsymbol{x}_n|\boldsymbol{Y}_n)d\boldsymbol{x}_n} \quad (6)$$

Equations 5 and 6 implement an online algorithm for backward inference in the sense that there is no need to store the time series of observations, $\boldsymbol{Y}_n$. In signal processing these updates are known as the gammma recursions [38] and it is these which we envisage could be implemented in the brain.

There is an alternative procedure for backward inference known as the beta recursions. We include this for completeness below, as there is an interesting connection to optimal control theory which we will also refer to in the discussion. However, the beta recursions require storage of the observations and so are not an online algorithm.

Alpha-Beta Decomposition

The distribution of hidden states $\boldsymbol{x}_n$ given an observed trajectory $\boldsymbol{Y}_1^N$ can be expressed using Bayes rule

$$\begin{aligned} p(\boldsymbol{x}_n|\boldsymbol{Y}_1^N) &= \frac{p(\boldsymbol{Y}_1^N|\boldsymbol{x}_n)p(\boldsymbol{x}_n)}{p(\boldsymbol{Y}_1^N)} \\ &= \frac{p(\boldsymbol{Y}_1^{n-1}|\boldsymbol{x}_n)p(\boldsymbol{Y}_n^N|\boldsymbol{x}_n)p(\boldsymbol{x}_n)}{p(\boldsymbol{Y}_1^N)} \\ &= \frac{p(\boldsymbol{Y}_1^{n-1},\boldsymbol{x}_n)p(\boldsymbol{Y}_n^N|\boldsymbol{x}_n)}{p(\boldsymbol{Y}_1^N)} \\ &= \frac{\alpha(\boldsymbol{x}_n)\beta(\boldsymbol{x}_n)}{p(\boldsymbol{Y}_1^N)} \end{aligned} \tag{7}$$

where $\alpha(\boldsymbol{x}_n) = p(\boldsymbol{Y}_1^{n-1},\boldsymbol{x}_n)$ and $\beta(\boldsymbol{x}_n) = p(\boldsymbol{Y}_n^N|\boldsymbol{x}_n)$. In offline applications, where an agent may store the time series of observations, the alpha and beta recursions can therefore be run in parallel. The alpha-beta algorithm is also therefore known as the parallel update [118]. The alpha and beta updates can then be combined to estimate the posterior probability of the hidden states given both past and future observations, as described above.

Beta recursions

Following on from the previous section we have

$$\beta(\boldsymbol{x}_n) = p(\boldsymbol{Y}_n^N|\boldsymbol{x}_n) \tag{8}$$

This can be computed recursively using

$$\beta(\boldsymbol{x}_n) = p(\boldsymbol{y}_n|\boldsymbol{x}_n)\int p(\boldsymbol{x}_{n+1}|\boldsymbol{x}_n)\beta(\boldsymbol{x}_{n+1})d\boldsymbol{x}_{n+1} \tag{9}$$

The beta recursions operate on future data and are the time-reversed equivalent of the alpha recursions. Under weak assumptions this is equivalent to Bellman's equation for recursively computing the optimal value of a state [119].
