# Supplementary material for: Forward and Backward Inference in Spatial Cognition
Source: PLoS Comput Biol. 2013 Dec 12;9(12):e1003383. doi: 10.1371/journal.pcbi.1003383 (PMC3861045; doi:10.1371/journal.pcbi.1003383)
Supplement: Text S3 — Shows how inference over inputs can be accommodated in the standard state-space framework by using an augmented model where the hidden states are an augmented vector comprising both the original states and the inputs. (PDF) [file pcbi.1003383.s003.pdf]

Text S3: Forward and Backward Inference in Spatial Cognition

Will D. Penny, Peter Zeidman and Neil Burgess

Augmented model

The model in equation 31 (main text) is equivalent to a state-space model without inputs but with an augmented state vector $\tilde{\boldsymbol{x}}_n = [\boldsymbol{x}_n, \boldsymbol{u}_n]^T$ where

$$\begin{aligned} \tilde{\boldsymbol{x}}_n &= \tilde{\boldsymbol{F}}_n \tilde{\boldsymbol{x}}_{n-1} + \tilde{\boldsymbol{z}}_n \\ \boldsymbol{y}_n &= \tilde{\boldsymbol{G}}_n \tilde{\boldsymbol{x}}_n + \boldsymbol{e}_n \end{aligned} \tag{1}$$

with $\tilde{\boldsymbol{z}}_n \sim N(\tilde{\boldsymbol{z}}_n; \boldsymbol{0}, \tilde{\boldsymbol{Q}})$ and $\boldsymbol{e}_n \sim N(\boldsymbol{e}_n; \boldsymbol{0}, \boldsymbol{R})$ and

$$\tilde{\boldsymbol{F}}_n = \begin{bmatrix} \boldsymbol{F}_n & \boldsymbol{H}_n \\ \boldsymbol{0} & \boldsymbol{0} \end{bmatrix} \tag{2}$$

$$\tilde{\boldsymbol{G}}_n = \begin{bmatrix} \boldsymbol{G}_n & \boldsymbol{0} \end{bmatrix} \tag{3}$$

and

$$\tilde{\boldsymbol{Q}} = \begin{bmatrix} \boldsymbol{Q}_x & \boldsymbol{0} \\ \boldsymbol{0} & \boldsymbol{Q}_u \end{bmatrix} \tag{4}$$

We assume the initial distributions over state and controls are Gaussian with means $\boldsymbol{m}_0$, $\boldsymbol{r}_0$ and covariances $\boldsymbol{P}_0$ and $\boldsymbol{B}_0$. The mean and covariance of the initial augmented state are therefore $\tilde{\boldsymbol{m}}_0 = [\boldsymbol{m}_0, \boldsymbol{r}_0]^T$ and

$$\tilde{\boldsymbol{P}}_0 = \begin{bmatrix} \boldsymbol{P}_0 & \boldsymbol{0} \\ \boldsymbol{0} & \boldsymbol{B}_0 \end{bmatrix} \tag{5}$$

We can therefore implement forward and backward inference as described in the main text but using the augmented quantities $\tilde{\boldsymbol{F}}_n$, $\tilde{\boldsymbol{G}}_n$, $\tilde{\boldsymbol{Q}}$ and $\tilde{\boldsymbol{P}}_0$ instead of $\boldsymbol{F}_n$, $\boldsymbol{G}_n$, $\boldsymbol{Q}$ and $\boldsymbol{P}_0$. Additionally, the $\boldsymbol{H}_n$ term in equation 23 (main text) is set to zero as the input dependence is already incorporated in $\tilde{\boldsymbol{F}}_n$. The desired control density is a Gaussian

$$p(\boldsymbol{u}_n | \boldsymbol{x}_1, \boldsymbol{Y}_N) = \mathsf{N}(\boldsymbol{u}_n; \hat{\boldsymbol{u}}_n, \hat{\boldsymbol{B}}_n) \tag{6}$$

and the quantities $\hat{\boldsymbol{u}}_n$ and $\hat{\boldsymbol{B}}_n$ from backward inference, can then be read off from the corresponding posterior estimates of the augmented states. In the absence of correlations between inputs and hidden states the update formulae for $\hat{\boldsymbol{u}}_n$ and $\hat{\boldsymbol{B}}_n$ have the simpler form shown in equation 34 in the main text.
