# Supplementary material for: Forward and Backward Inference in Spatial Cognition
Source: PLoS Comput Biol. 2013 Dec 12;9(12):e1003383. doi: 10.1371/journal.pcbi.1003383 (PMC3861045; doi:10.1371/journal.pcbi.1003383)
Supplement: Text S4 — Presents an alternative formulation of equation 24 in the main text, showing how the relative contribution of path integration and sensory cues, to the estimation of hidden states, is a function of their relative precision [59], [120]. (PDF) [file pcbi.1003383.s004.pdf]

Text S4: Forward and Backward Inference in Spatial Cognition

Will D. Penny, Peter Zeidman and Neil Burgess

Combining path integration with sensory input

Several studies have explored how head direction cells respond when information from landmark sources conflicts with self-motion signals [59]. Most studies have found that when well-established and prominent visual cues are placed in conflict with self-motion cues, the spatial information derived from visual landmarks prevails. During spatial localisation these two types of information are optimally combined using Bayes rule.

After a path integration update, the uncertainty on the state variables is $\boldsymbol{Q}_n$ and the uncertainty of the sensory information is $\boldsymbol{R}$. The relative values of $\boldsymbol{R}^{-1}$ and $\boldsymbol{Q}_n^{-1}$ then determine which source dominates, with the higher precision source primarily determining the subsequent state estimate. This can be seen more easily by writing equation 24 in the main text in a different but mathematically equivalent form

$$\begin{aligned} \boldsymbol{P}_n^{-1} &= \boldsymbol{G}^T \boldsymbol{R} \boldsymbol{G} + \boldsymbol{Q}_n^{-1} \\ \boldsymbol{m}_n &= \boldsymbol{P}_n (\boldsymbol{G}^T \boldsymbol{R}^{-1} \boldsymbol{y}_n + \boldsymbol{Q}_n^{-1} \boldsymbol{\mu}_n) \end{aligned} \quad (1)$$

If, for example, path integration is exceptionally noisy then $\boldsymbol{Q}_n^{-1} = 0$ and $\boldsymbol{m}_n = \boldsymbol{W} \boldsymbol{y}_n$ where $\boldsymbol{W} = (\boldsymbol{G}^T \boldsymbol{R} \boldsymbol{G})^{-1} \boldsymbol{G}^T \boldsymbol{R}^{-1}$ is the weighted least squares operator. That is, state variables are determined solely by sensory input.

Relative precisions of sensory input

The model we have described in the main text assumes that the different modalities are independent given the state. This gives the observation noise covariance a block diagonal structure $\boldsymbol{R} = \mathsf{blkdiag}(R_0, R_t, \boldsymbol{R}_v)$. The precision will have a similar structure $\boldsymbol{R}^{-1} = \mathsf{blkdiag}(R_0^{-1}, R_t^{-1}, \boldsymbol{R}_v^{-1})$. The values of the covariance parameters R_0, R_t,$\boldsymbol{R}_v$ therefore determine the weight $\boldsymbol{W}$ that is given to the different sensory modalities. For example, setting R_t to a very high value effectively switches off somatosensory input.

During the experiments in this paper we found, for example, that somatosensory inputs are only useful during route and motor planning. Somatosensory input is necessary here so that the route plans avoids walls in the environment. However, somatosensory input was found to be deleterious for the tasks of localisation and decision making. In the brain, the precision of sensory input is hypothesised to be modified by changing its gain. This could happen either by changes in synaptic plasticity or in the temporal synchronization of input activity [120]. Thus, we expect that the different modes of computation (localisation, route planning etc), are accompanied by large scale changes in effective connectivity in the brain. This point is addressed further in the discussion (see 'localisation' under 'Neuronal Implementation').
